# Dual Regulation on Structure‐Interface Enables Coal‐Tar‐Pitch‐Based Hard Carbon Anodes with High Rate and Storage Performance for Sodium Ion Batteries

**DOI:** 10.1002/advs.202515146

**Published:** 2025-10-05

**Authors:** Xinmeng Xu, Kun Wang, Beibei Han, Jianke Li, Baigang An, Chengguo Sun, Guiying Xu, Zewei Li, Wenwu Zhang, Zhenbo Wang, Weimin Zhou

**Affiliations:** ^1^ Key Laboratory of Energy Materials and Electrochemistry Research Liaoning Province University of Science and Technology Liaoning No. 189, Qianshan Middle Road, Lishan District, Anshan Liaoning 114051 P. R. China; ^2^ State Key Laboratory of Space Power‐Sources MIIT Key Laboratory of Critical Materials Technology for New Energy Conversion and Storage MOE Engineering Research Center for Electrochemical Energy Storage and Carbon Neutrality in Cold Regions School of Chemistry and Chemical Engineering Harbin Institute of Technology Harbin 150001 P. R. China; ^3^ Key Laboratory of Advanced Fuel Cells and Electrolyzers Technology of Zhejiang Province Ningbo Institute of Materials Technology and Engineering Chinese Academy of Sciences No. 1219 Zhongguan West Road Ningbo Zhejiang 315201 P. R. China; ^4^ Weida New Material Technology Co., Ltd. Jixi 158100 P. R. China; ^5^ Haicheng Shenhe Technology Co., Ltd Haicheng 114213 P. R. China

**Keywords:** anode materials, coal tar pitches, hard carbons, sodium‐ion batteries (SIBs), structure‐interface

## Abstract

Coal‐tar‐pitches‐based hard carbons (HCs) are regarded as promising anode materials for sodium‐ion batteries (SIBs). However, designing optimal microstructures and surface chemical states of carbon anodes to enhance Na^+^ diffusion kinetics remains a key challenge for superior sodium storage. Herein, a novel strategy of molecular crosslinking‐coupled chemical vapor deposition (CVD) with further post‐heat treatment is proposed. This approach utilizes molecular cross‐linking to restrict the strong *π*–*π* interactions among the aromatic rings of polycyclic aromatic hydrocarbons (PAHs) in the coal tar pitches (CTPs) when simultaneously introducing the developed pore structures with large interlayer spacing into the carbon matrix. The surface carbon coatings by the CVD method can facilitate the transition from the open pores to closed pores. The subsequent post‐treatment can effectively regulate the surface chemistry of carbon anodes. Benefiting from the dual regulations on structure‐interface, the optimized HPCV5‐1200 exhibited a high initial cycle efficiency (ICE) of 91.6% and 320.2 mAh g^−1^ after 300 cycles at 0.2 A g^−1^. Moreover, the HPCV5‐1200 demonstrated the superior rate capacity (112.6 mAh g^−1^ at 10 A g^−1^) with 53.1% of reversible capacity below 0.1 V. Furthermore, the Na_3_V_2_(PO_4_)_3_ (NVP)//HPCV5‐1200 full cell exposes the high energy density of 233.5 Wh kg^−1^, with desirable cycling stability and rate performance.

## Graphical abstract

This study utilizes coal tar pitch (waste product generated during the coal conversion process) as a raw material. The core concept of synergistic “structure‐interface” regulation, an innovative system from molecular design to microcrystalline optimization, pore reconstruction, and interface regulation is proposed. The fabricated HPCV5‐1200 exhibits high initial coulombic efficiency (ICE) (91.6%) and excellent rate performance.

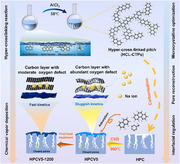

## 1 Introduction

SIBs are considered the most promising alternative to lithium‐ion batteries (LIBs) due to the abundant reserves and low cost of sodium resources.^[^ ^1^, ^2^, ^3^ ^]^ Nevertheless, the rational design of electrodes with excellent sodium storage performance and low‐cost still remains a key challenge for the commercial application of SIBs. Although numerous materials such as carbon‐based materials, alloys, metal oxides, and small organic molecules have been applied to SIB negative electrodes,^[^ ^4^, ^5^ ^]^ hard carbons (HCs) with randomly oriented carbon layers and enlarged interlayer spacing are regarded as one of the most promising anode materials for practical applications, owing to their high sodium storage capacity and low working voltage (vs Na^+^/Na).^[^ ^6^ ^]^

HCs can be obtained by the pyrolysis of thermosetting resins, organic macromolecules, biomass precursors under a high carbonization temperature (1300 °C).^[^ ^7^, ^8^, ^9^ ^]^ However, the low yield of carbon precursors during high‐temperature pyrolysis results in significantly increased production costs, severely hindering their practical applications. Therefore, the preparations of economically efficient HCs precursors and the exploration of their excellent electrochemical performance are becoming hot research topics.

CTPs as an industrial product are mainly composed of PAHs, which ensures a high carbon yield after their pyrolysis. However, direct pyrolysis of CTPs undergoes a liquid‐phase carbonization process due to their high hydrogen content and strong *π–π* interactions between aromatic molecules, ultimately resulting in the formation of highly graphitized soft carbon with small layer spacing.^[^ ^10^ ^]^ This microstructural feature is detrimental to Na^+^ storage performance, leading to low reversible capacity and negligible plateau capacity. In this respect, numerous studies have demonstrated that the incorporation of oxygen‐crosslinked structures into CTPs can significantly facilitate the transformation of their microstructures from an ordered graphite structure to disordered phases with closed pore structures.^[^ ^11^, ^12^, ^13^ ^]^ However, although the oxygen crosslinker altered the microcrystalline structure of carbon, it concurrently introduced a substantial number of oxygen defects within the carbon framework. These defects may disrupt the continuous sodium ion diffusion channels and diminish the electrical conductivity of the electrode materials, thereby leading to difficulties in further improving the high‐rate sodium storage performance of the materials.^[^ ^14^ ^]^ Therefore, a novel efficient cross‐linking method to enhance the comprehensive sodium storage performance of CTPs‐based carbon (CTPC) materials needs to be proposed urgently.

Furthermore, recent studies have demonstrated that engineering HCs with a closed‐pore structure can effectively reduce the specific surface area, thus mitigating irreversible reactions associated with open pores and surface defects. This approach not only improves the ICE but also enhances the sodium storage capacity and cycling stability. One effective strategy involves depositing a carbon layer on the surface of porous carbon via the CVD method, which promotes the conversion of open pores into closed pores. For example, Liu et al carried out the CVD method using the pitch as the carbon source. And propose that the pitch‐derived soft carbon coating layer can facilitate the transition from exposed to closed pores.^[^ ^10^ ^]^ Similarly, Chen et al. precisely regulated the growth and thickness of the surface carbon layer by adjusting the amount of pitch deposited, and systematically investigated the influence of carbon layer thickness on sodium storage performance.^[^ ^15^ ^]^ However, it is noteworthy that the aforementioned studies often overlook the regulation of defects in the surface carbon layer and its potential impact on the electrode/electrolyte interface.

From the perspective of the electrolyte/electrode interface, the inorganic‐rich and thin solid electrolyte interphase (SEI) structure can reduce the diffusion length of Na^+^ and accelerate sodium ion transport, while enhancing the cycling stability of the material.^[^ ^16^, ^17^, ^18^ ^]^ The concept of weakly solvating electrolytes (WSE) has gained significant traction in the development of inorganic‐rich and thin SEI on anodes. This is accomplished through the optimization of the electrolytes, which involves selecting suitable solvents and additives, modifying the composition and concentration of the electrolyte, and balancing solvation capabilities.^[^ ^19^, ^20^, ^21^, ^22^ ^]^ Unfortunately, most WSE applications emphasis the intrinsic properties of electrolytes while ignoring the electrode/electrolyte interface that affects the solvated sheath, which limits the effect of forming an ideal SEI film in real battery systems.

Herein, our studies propose a structure‐interface synergistic engineering strategy based on the triple synergistic mechanisms of “microcrystalline optimization – pore reconstruction – interface regulation” to design HCs anode materials with high electrochemical performance. First, the cross‐linking of PAHs in CTPs was successfully achieved through the Friedel‐Crafts·alkylation reaction. Consequently, the microstructures of CTPC were effectively transformed from an ordered graphite‐like structure to disordered structures with open pores. Then, to reduce the specific surface area of samples, a CVD method was employed to form multilayer graphene structures on the surface of the CTPC. Finally, the overall material structure and surface chemical properties were reorganized by a post‐heat treatment to obtain a weakly solvating interface for a thin and inorganic‐rich SEI layer to enhance the Na^+^ diffusion kinetics from the surface to the bulk phase. Notably, the optimized sample (HPCV5‐1200) shows prominent sodium‐ion storage performance, a high ICE of 91.6%, and a superior reversible Na^+^ storage capacity of 320.2 mAh g^−1^ at 0.2 A g^−1^ after 300 cycles with a dominant plateau region capacity. In particular, the obtained HPCV5‐1200 anode also exhibits a superior rate performance of 112.6 mAh g^−1^ at 10 A g^−1^ with 53.1% of reversible capacity below 0.1 V. This work provides new insights into the realization of microstructure and interfacial regulation for high‐performance sodium storage in CTPs‐based anode materials.

## 2 Results and Discussion

**Figure 1:**
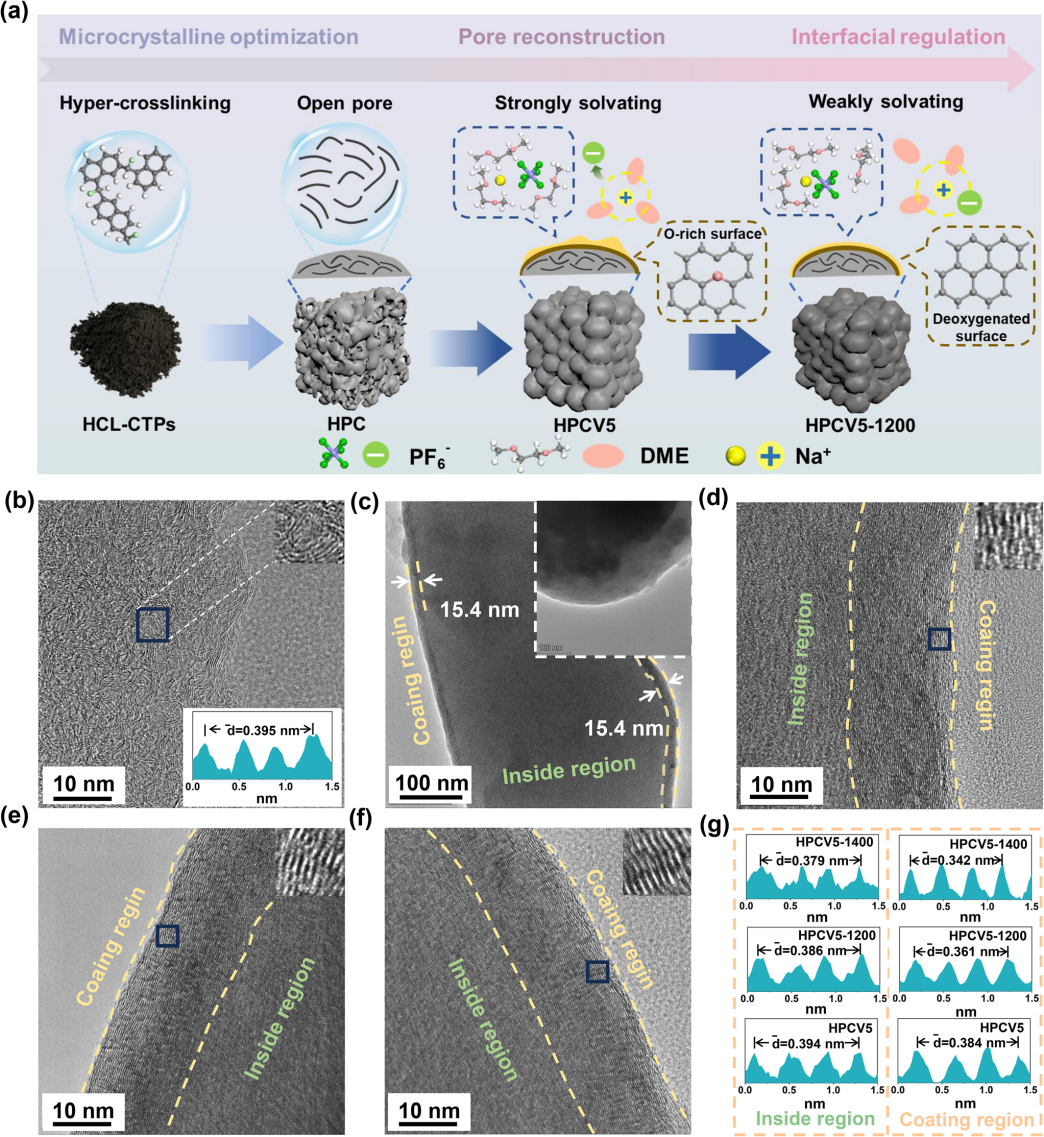
a) Schematic diagram of the preparation of the samples. b) HRTEM image of HPC. c) is the TEM image of HPCV5‐1200, and the inset shows the TEM image of HPC. HRTEM images of d) HPCV5, e) HPCV5‐1200, and f) HPCV5‐1400. g) CTPs‐based HCs with the corresponding fast Fourier transform (FFT) patterns.

**Figure** **1**a exhibits the synthesis procedure of CTPs‐based HCs with large interlayer spacing and low‐porosity structures. In a typical procedure, the PAHs in CTPs were hyper‐crosslinked by an aluminum chloride catalyst to form a large 3D cross‐linked aromatic network structure to obtain hyper‐crosslinked CTPs (HCL‐CTPs). Then, HCL‐CTPs were carbonized at 1200 °C under an Ar atmosphere to get HCL‐CTPs‐based hard carbon (HPC). In order to reduce the specific surface area of HPC, CTPs were selected as a carbon source for the CVD process. During the CVD procedure, CTPs may decompose and release volatile compounds such as an thracene, naphthalene, phenanthrene, phenol, and bisphenol A.^[^ ^15^, ^23^ ^]^ These volatile compounds are carried by the inert gas flow and deposited onto the surface of HPC, forming a surface carbon coating layer. Subsequent post‐heat treatment can further regulate the bulk structure and surface defect concentration, thereby optimizing the electrode/electrolyte interface, reducing the Na^+^ diffusion energy barrier, and enhancing the Na^+^ storage capacity of the optimized HPCV5‐1200.

The chemical states of HCL‐CTPs are surveyed by XPS first. As shown in the C 1s spectra of HC‐CTPs (Figure S2, Supporting Information), the emergence of a new peak at 286.7 eV is assigned to the C─Cl bond.^[^ ^24^, ^25^ ^]^ This assignment is further corroborated by the characteristic peaks of the Cl 2p spectrum at 200.0 and 201.6 eV.^[^ ^26^, ^27^ ^]^ As shown in Figure S3 (Supporting Information), the (002) peak of HCL‐CTPs exhibits significant broadening and shifts to a lower angle. These findings collectively suggest that the substitution of hydrogen atoms in PAHs with chlorine from trichloromethane (CHCl_3_) results in intermolecular cross‐linking among PAHs, thereby disrupting their parallel *π–π* stacking arrangement.

Figure S4 (Supporting Information) shows the digital images of the carbonization process of CTPs and HCL‐CTPs at different temperatures. As illustrated in Figure S4 (Supporting Information), the CTPs will be transformed into the liquid state when heating to 300 °C, whereas the HCL‐CTPs consistently remain in solid powder form. Even when heated to 1200 °C, no significant bonding phenomenon was observed among the powder particles, confirming that the C─Cl cross‐linking between the PAHs successfully suppressed the thermal motion of the aromatic molecules, thereby shifting CTPs from a thermoplastic to a thermosetting state. The obtained carbon yield of 57.4% for HCL‐CTPs at the pyrolysis temperature is higher than the CTPs of 33.1% at 1000 °C, also indicating that HCL‐CTPs exhibit better thermal stability (Figure S5, Supporting Information). Finally, the C─Cl cross‐linking in the PAHs was further verified by the solid‐state ^13^C CP/MAS NMR (Figure S6, Supporting Information).

High‐resolution transmission electron microscopy (HRTEM) measurements were performed to characterize the microcrystalline structure of the prepared carbon materials. Figure 1b shows that the HPC exhibits the highly disordered structure and large interlayer spacing (0.395 nm), implying that the cross‐linking reaction inhibits the graphitization and causes randomly distributed turbostratic carbon layers. As shown in Figures 1c and S7 (Supporting Information), a uniform carbon coating layer (≈15.4 nm thick) was successfully deposited on the HPC surfaces during the CVD process. The rough surface of HPC exhibits granular protrusions, whereas the surfaces of HPCV5, HPCV5‐1200, and HPCV‐1400 materials appear significantly smoother. This morphological difference suggests the successful formation of a uniform carbon coating on the HPC (Figure S8, Supporting Information). It is worth noting that the carbon coating is in direct contact with the electrolyte, the microstructure and surface chemical properties of carbon coatings have a significant impact on the formation of SEI films, which is also crucial for enhancing the electrochemical performance of carbon anode materials.^[^ ^17^, ^28^, ^29^ ^]^

As illustrated in Figure 1d–g, the distinct carbon layer structures are observed both on the surface and within the particles. The coated carbons on the surfaces of HPCV5 exhibit a short‐range ordered structure with an expanded interlayer spacing (d = 0.384 nm), which belongs to typical microcrystalline structures of soft carbons. With an increase in post‐heat treatment temperature, the d‐spacing of the coating layers decreased to 0.361 nm (HPCV5‐1200) and 0.342 nm (HPCV5‐1400), exhibiting a more ordered graphite‐like microcrystalline structure. In contrast, the internal carbon matrix exhibited a highly disordered structure, characterized by carbon layers with expanded interlayer d‐spaces of 0.394, 0.386, and 0.379 nm for HPCV5, HPCV5‐1200, and HPCV5‐1400, respectively. These values are significantly larger than the typical graphite interlayer distance of 0.37 nm, indicating a substantial degree of structural disorder within the carbon matrix. The distinct structural differences between the internal carbon matrix and the surface carbon coating demonstrate that the hyper‐crosslinking reaction effectively constructs a rigid non‐graphitized carbon skeleton within the matrix, while the soft carbon coating undergoes significant structural reorganization during high‐temperature annealing, resulting in tunable surface properties.

**Figure 2:**
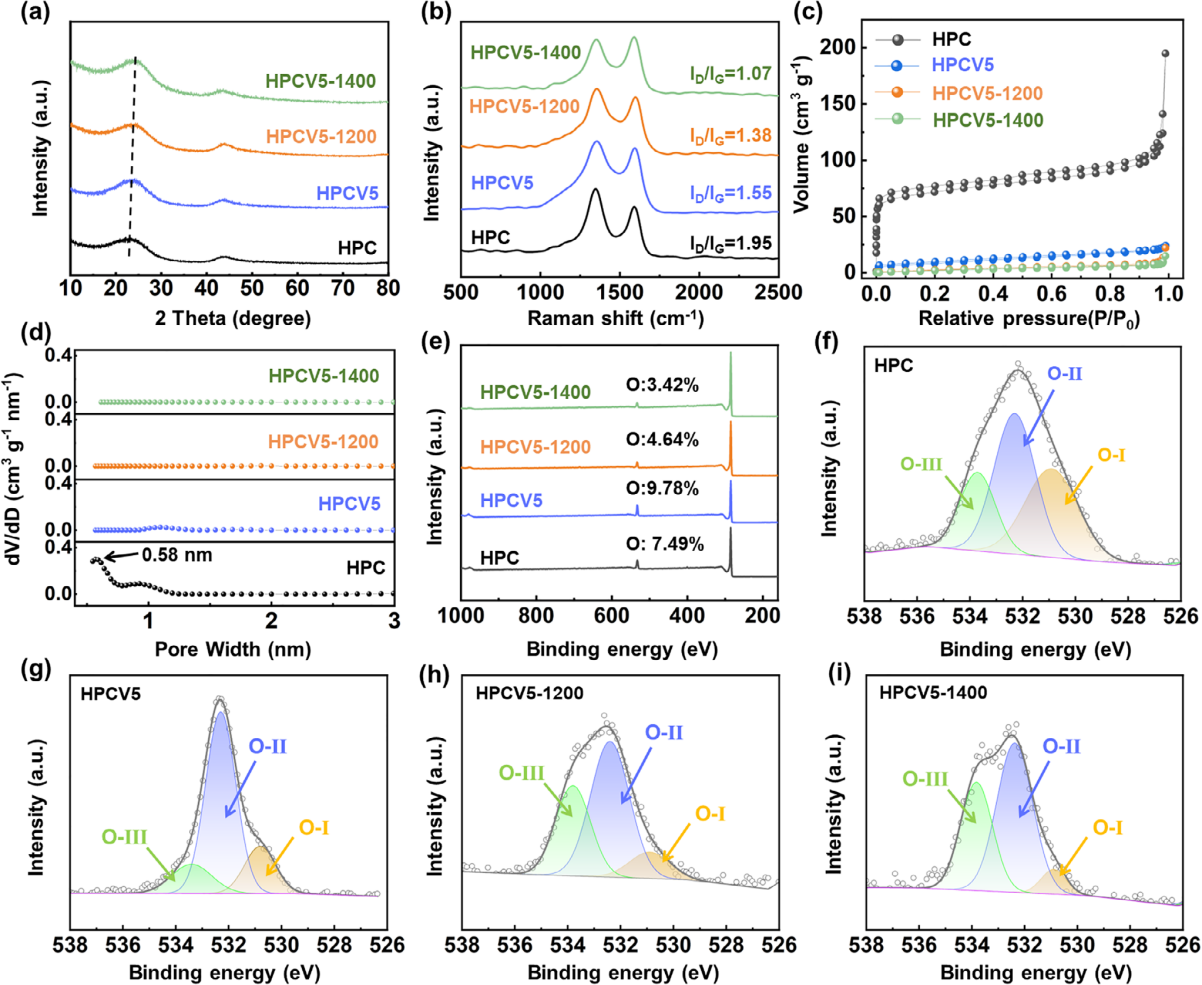
a) XRD patterns, b) Raman spectra, and c) N_2_ adsorption–desorption isothermal curves of the materials. d) The corresponding pore size distributions of the materials. e) XPS survey spectra and the high‐resolution O1s spectra of f) HPC, g) HPCV5, h) HPCV5‐1200 and i) HPCV5‐1400.

The microstructures of carbon materials were investigated by X‐ray diffraction (XRD) and Raman spectroscopy. It can be seen that two broad peaks ≈23° and ≈44° are contributed to the (002) and (100) diffraction peaks of amorphous carbon materials (**Figure** **2**a).^[^ ^30^, ^31^ ^]^ From HPC to HPCV5‐1400, the (002) diffraction peaks shift to higher angles, indicating an increase in graphitization degree. Meanwhile, the interlayer spaces of HPC, HPCV5, HPCV5‐1200, and HPCV5‐1400 are calculated as 0.398, 0.388, 0.375, and 0.368 nm, respectively. Besides, Raman data indicated that the *I_D_/I_G_* values of HPC, HPCV5, HPCV5‐1200, and HPCV5‐1400 decreased from 1.95 to 1.07 (Figures 2b; S9, Supporting Information), revealing that the surface defects and disorder degree of the materials have been significantly reduced,^[^ ^32^, ^33^ ^]^ which is also consistent with the results of XRD analyses.

The specific surface areas and pore size distributions of the obtained carbons were analyzed through nitrogen adsorption and desorption measurements. During pyrolysis, the open pore structure formed by the small gas molecules released from the degradation of cross‐linked polymers in PAHs results in a high N_2_ absorption capacity of HPC. This is characterized by a high specific surface area (S_BET_) of 271.3 m^2^ g^−1^ and a large total pore volume (V_t_) of 0.301 cm^3^ g^−1^ (Figure 2c). As shown in Figure 2d, the pore size distribution of the HPC is mainly centered at 0.58 nm, which facilitates the adsorption of small‐molecule gases such as benzene, phenol, and anthracene (common volatile compounds generated from CTPs during CVD processing) onto the micropore surfaces of HPC. By the catalytic effect of the pore wall,^[^ ^34^ ^]^ the adsorbed species can pyrolyze into few‐layer graphene and deposit sequentially layer‐by‐layer onto the pores wall, blocking the transmission path of subsequent gases and limiting the gas diffusion, thus generating some closed micropores in HPC and reducing the open pore structure.^[^ ^34^ ^]^ After CVD treatment, the results show that the specific surface area of HPCV5 decreases to 30.6 m^2^ g^−1^, indicating a sharp decrease in the number of open‐pore structures. Notably, after further post‐heat treatment, the specific surface areas of HPCV5‐1200 and HPCV5‐1400 decrease to 9.6 and 8.0 m^2^ g^−1^, respectively. This reduction of surface areas may be attributed to the densification of carbon layers caused by orderly stacking and migration of the surface carbon layers at high‐temperature,^[^ ^35^, ^36^ ^]^ which can facilitate the limitation of SEI generation and enhance the ICE value during the initial charge/discharge cycle.

In addition, small‐angle X‐ray scattering (SAXS) was employed to evaluate the closed pore structure. As shown in Figure S10 (Supporting Information), all samples exhibit broad humps around the scattering vector (Q) of 0.1 Å^−1^, further confirming the presence of closed micropores in HPCV materials.^[^ ^37^, ^38^ ^]^ Generally, graphite materials with an ideal layered structure exhibit the highest true density (2.26 g cm^−3^) among carbon materials.^[^ ^39^ ^]^ The closed pore volume of porous carbon can be calculated by the equation: V_closed pore_ = 1/ρ_ture_−1/2.26, where ρ_true_ is the true density of the material.^[^ ^40^ ^]^ As shown in Table S1 (Supporting Information), the closed pore volume of HPCV5 and HPCV5‐X exceeds that of HPC (0.113 cm^3^ g^−1^), further substantiating the transition of open pores to closed pores during the CVD process.

The surface chemical states of HPC, HPCV5, and HPCV5‐X were explored by X‐ray photoelectron spectroscopy (XPS) measurements. Figure 2e shows distinct C 1s (284.8 eV) and O 1s (532.1 eV) peaks in the XPS spectrum, with no detectable Cl 2p signal (≈200 eV),^[^ ^27^, ^41^, ^42^ ^]^ indicating thermal decomposition of C─Cl bonds into volatile small molecules during carbonization. On the basis of XPS measurements, it is aware of that the oxygen content of HPC is 7.49 at.%. The oxygen content of HPCV5 increased to 9.78% following the deposition of the carbon coating via the CVD method, in which CTPs were used as a carbon source at the relatively low temperature of 1000 °C. As the temperature increased during post‐heat treatment, the oxygen content in HPCV5‐1200 and HPCV5‐1400 significantly decreased to 4.64% and 3.42%, respectively. As shown in Table S2 (Supporting Information), it is distinct that the oxygen content of the prepared HPCV5 material slightly increased, after the HPC material was coated with the carbons. However, with increasing carbonization temperatures, the oxygen concentrations show a decreasing trend. In order to better determine the existence types of oxygen‐containing functional groups in the HPC and HPCV materials, the O 1s spectrum can be divided into O‐I peaks (533.7 eV), O‐II peaks (532.2 eV), and O‐III peaks (531.1 eV).^[^ ^12^, ^22^ ^]^ The O‐I peak corresponds to carboxyl and ester groups (─COOH, ─COOR), the O‐II peak represents ether and hydroxyl groups (─C─O─C, ─COH), while the O‐III peak is attributed to carbonyl groups (C═O). All samples show the dominated O‐II peak, indicating the presence of abundant C─O─R functional groups. By the FT‐IR measurements(Figure S11, Supporting Information), the sharp peak attributing to the ─C─O─C groups of HPC, HPCV5, HPCV5‐1200, and HPCV5‐1400 was observed at ≈1100 cm^−1^.^[^ ^12^, ^41^ ^]^ Combined with the XPS results, it is considerable that the presence of ─C─O─C dominated oxygen defects is observed in HPC, HPCV5, HPCV5‐1200, and HPCV5‐1400, respectively. Although Titirici et al. have demonstrated that oxygen defects dominated by ─C─O─C exhibit high adsorption energy toward sodium ions, thereby contributing to enhanced Na^+^ storage capacity.^[^ ^43^ ^]^ However, elevated concentrations of oxygen defects may capture Na^+^ ions, thereby disrupting the continuous transfer of Na^+^ and compromising the rapid transport kinetics within carbon layers.^[^ ^15^ ^]^ In addition, the influence of oxygen‐rich interface on the formation of the SEI is often underestimated. Collectively, to the best of our knowledge, no systematic studies have comprehensively addressed these multifaceted effects of oxygen defects on the electrochemical performance of carbon anodes.

The sodium storage performance of the carbon samples was evaluated through galvanostatic charge/discharge (GCD) curve measurement. The charge/discharge curves of all samples consisted of a low‐voltage plateau region (< 0.1 V) and a high‐voltage sloping region (> 0.1 V). Figure S12 (Supporting Information) illustrates the charge/discharge curves of HPCV3, HPCV5, and HPCV7 with different contents of the CTP‐based soft carbon layers on their surfaces. HPC exhibits the reversible Na^+^ storage capacity of was 236.7 mAh g^−1^ at a current density of 50 mA g^−1^ with the ICE of 80.9% at first cycle. Compared with HPC, HPCV3, HPCV5, and HPCV7 exhibit improvements in both reversible capacity and ICE. Notably, HPCV5 delivers a higher reversible Na^+^ storage capacity (247.8 mAh g^−1^) and ICE (85.8%) at the first cycle. However, HPCV7 shows a decrease in reversible capacity (189.9 mAh g^−1^) and ICE (78.7%). To explore the reasons for this performance variation, the microstructural evolution of the materials was further investigated. As shown in Figures S14 and S15 (Supporting Information), the carbon coating on HPCV3 exhibits discontinuous carbon coatings with a thickness of ≈7.4 nm. Compared with the HPCV5 (30.6 m^2^ g^−1^), this results in a significantly higher specific surface area for HPCV3 (139.09 m^2^ g^−1^). In contrast, the increased content of CTPs‐based volatiles caused a dramatic increase in the carbon coating thickness on HPCV7 to 31.1 nm. Although the specific surface area of HPCV7 further decreased to 13.3 m^2^g^−1^, its ICE and reversible capacity are lower than those of HPCV5. This indicates that the excessive thickness of the carbon coating hinders the Na^+^ diffusion from the electrode/electrolyte interface to the internal carbon matrix, resulting in additional irreversible capacity and a degradation of electrochemical performance.^[^ ^15^ ^]^ These characterization results reveal that controlling the content of volatiles of CTPs in CVD processes enables the tailored regulation of the carbon coating thickness, thereby optimizing the Na^+^ storage performance of carbon materials.

**Figure 3:**
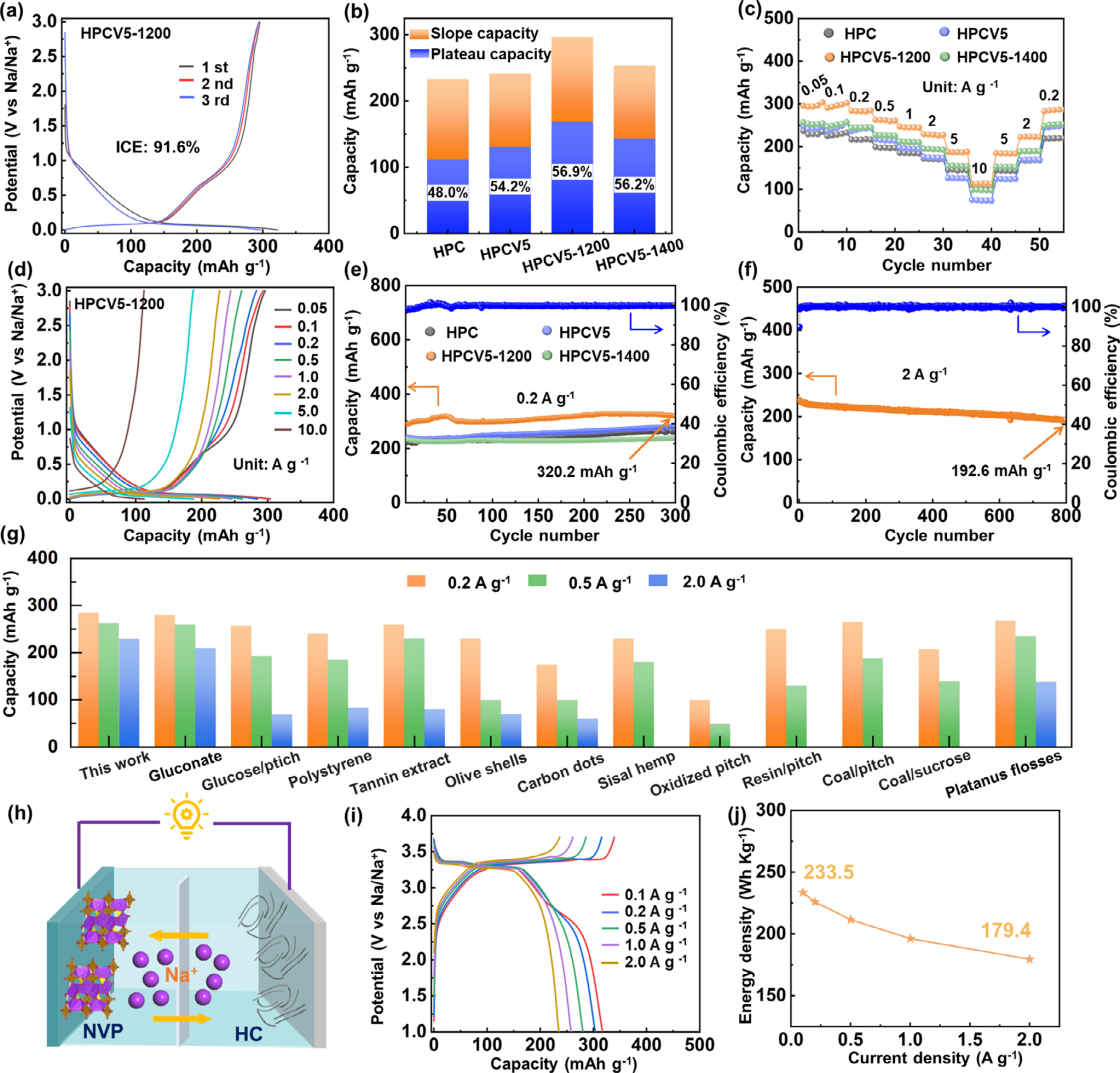
a) The discharge‐charge profiles of HPCV5‐1200. b) The plateau region capacity and sloping region capacity comparison in various carbon samples (based on the third charge curve). c) Rate performance at different current densities. d) Galvanostatic charge–discharge curve of HPCV5‐1200 at different current densities. e) Cycle performance at 0.2 A g^−1^. f) Cycle performance at 2 A g^−1^. g) Comparison of the capacities for HPCV5‐1200 in this work with HCs reported in literature. h) The schematic illustration of the full configuration, where the HPCV5‐1200 anode is coupled with the NVP cathode. i) Charge/discharge curves of full cell. j) The energy density of full cell at different current density.

Interestingly, after post‐heat treatment to adjust the microstructure and surface oxygen defect concentration of the HPCV5 material, the storage capacity of HPCV5‐1200 increased to 295.5 mAh g^−1^, and the ICE also rose to 91.6% at the first cycle with a current density of 0.05 A g^−1^ (**Figure** **3**a). It is attributed to that HPCV5‐1200 possesses the small specific surface area, appropriate interlayer spacing and low oxygen defect concentration. However, with the further increase of the carbonization temperature, it is observed that both the initial capacity and ICE of the HPCV5‐1400 material decreased (Figure S16, Supporting Information), which might be due to the poor reversibility of Na^+^ intercalation/extraction caused by the small interlayer spacing.^[^ ^44^ ^]^ Figure 3b describes the capacity contributions from the plateau region (< 0.1 V) and the slope region (> 0.1 V) in the discharge curve during the third cycle. It can be seen that the HPCV5, HPCV5‐1200, and HPCV5‐1400 exhibit significantly higher plateau capacity ratios than the HPC. Among them, HPCV5‐1200 demonstrates the highest plateau capacity contribution (56.9%) with a plateau capacity of 168.5 mAh g^−1^. Figure 3c shows the rate performance of different samples, and the HPCV5‐1200 have the highest reversible specific capacities of 302.8, 302.2, 284.5, 260.6, 244.1, 227.1, 187.6, 147.6, and 112.5 mAh g^−1^ after five cycles at current densities of 0.05, 0.1, 0.2, 0.5, 1, 2, 5, and 10 A g^−1^, respectively. Even at 10 A g^−1^, the plateau capacity is still 59.8 mAh g^−1^, accounting for 53.1% of the total capacity (Figure 3d), indicating that the HPCV5‐1200 shows the rapid Na^+^ transfer kinetics. In contrast, the plateau capacity of HPCV5 almost completely disappeared at 10 A g^−1^ (Figure S17, Supporting Information), indicating the sluggish Na^+^ transport kinetics in the plateau‐voltage region, which may be due to the high concentration of oxygen defects inhibiting the rapid Na^+^ diffusion in the HPCV5 electrode. Therefore, it is essential to balance the physical parameters of the carbon interlayer spacing and surface oxygen defect concentration in the material to synergistically enhance the rapid transfer of electrons/ions from the surface to the bulk phase, thereby achieving optimal overall electrochemical performance.

Figure 3e illustrates the cycling performance of all carbon materials at 0.2 A g^−1^. After 300 cycles, the initial reversible capacity of HPCV5‐1200 is 320.2 mAh g^−1^, and a slight increase in capacity is observed during the charge/discharge cycles, which can be attributed to the activation process.^[^ ^15^ ^]^ Moreover, even after 800 cycles at a high current density of 2 A g^−1^, the HPCV5‐1200 electrode retains a high specific capacity of 192.6 mAh g^−1^, demonstrating its exceptional cycling stability. Furthermore, as shown in Figure 3g and Table S3 (Supporting Information), HPCV5‐1200 exhibits superior electrochemical storage performance compared to previously reported HCs anode materials. Finally, a full cell was assembled by using the HPCV5‐1200 anode and the NVP cathode (Figure 3h). The mass ratio of m_HPCV5‐1200_: m_NVP_ = 1:3 was adopted to ensure the performance of the full cells (HPCV5‐1200//NVP), achieving a high average operating voltage of 3.24 V with an energy density of 233.5 Wh kg^−1^ (Figure 3i). HPCV5‐1200//NVP still has good cycling stability after cycling 100 times at a current density of 2 A g^−1^ (Figure S18, Supporting Information), indicating that HPCV5‐1200 has great application potential in the actual SIBs. In addition, the full cell (HPCV5‐1200//NVP) exhibits the superior energy densities of 233.5, 225.9, 211.5, 196.2, and 179.4 Wh kg^−1^ after five cycles at current densities of 0.1, 0.2, 0.5, 1.0, and 2.0 A g^−1^, respectively (Figure S19, Supporting Information).

To gain insight into the charge storage behavior of fabricated materials, CV measurements were performed for HPC, HPCV5, HPCV5‐1200, and HPCV5‐1400 as shown in Figure S20 (Supporting Information). The irreversible reduction peaks ≈0.3–1.0 V in the first cycle correspond to the decomposition of the electrolyte to form the SEI film.^[^ ^11^, ^13^ ^]^ Among them, the irreversible reduction peak area of the first cycle for HPC is significantly larger than that of the other materials, indicating that its large specific surface area leads to the formation of more SEI films and a lower ICE. After the first cycle, the redox peaks at 0.1 V and the broad 0.1–2 V hump in CV curves directly correspond to the plateau and sloping regions in the charge/discharge profiles, respectively. Furthermore, the CV curves of all materials almost overlap in 2nd and 3rd cycles, demonstrating the good reversibility of the electrochemical reactions.

**Figure 4:**
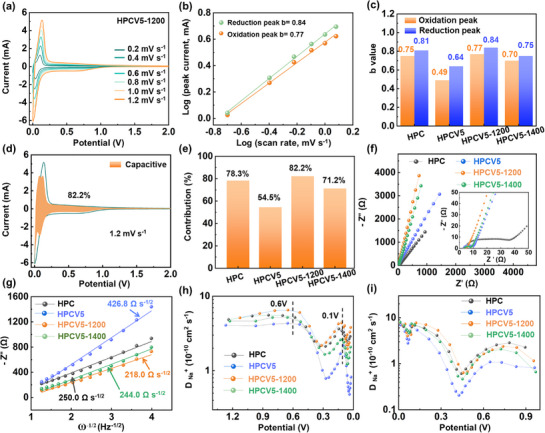
a) CV profiles of HPCV5‐1200 at different scan rates from 0.2 to 1.2 mV s^−1^ and b) the corresponding correlations between peak current (i) and scan rate (mV s^−1^). c) b value of all samples. d) CV curves displaying the capacitive contribution to the total current at 1.2 mV s^−1^ of HPCV5‐1200. e) Capacitive contribution of all samples at 1.2 mV s^−1^. f) EIS of the samples. g) Illustrations of relationships between Z′ and ω^−1/2^ in the low‐frequency region. Sodiumion diffusion coefficients at h) sodiation and i) desodiation process estimated from GITT curves.

To further investigate the kinetic property of the fabricated materials, CV curves at different scan rates were recorded in **Figures** **4**a and S21 (Supporting Information). In general, the b values can be calculated by the relationships between scan rates and currents (*i* = *av^b^*).^[^ ^7^, ^8^ ^]^ Generally, a b value close to 0.5 indicates diffusion‐controlled electrochemical reaction with slow Na^+^ storage kinetics, while a value close to 1.0 corresponds to dominant capacitive behavior with fast Na^+^ storage kinetics. The CV curves of HPC, HPCV5, HPCV5‐1200, and HPCV5‐1400 show a linear relationship between log(*υ*) and log(*i*) as shown in Figures 4b and S22 (Supporting Information), and the corresponding *b* values of all samples are shown in Figure 4c. By contrast, the *b* values corresponding to oxidative and reductive peaks of HPC are 0.75 and 0.81, respectively. Following carbon coating, the *b* values of HPCV5 decreased significantly to 0.49 (oxidation peak) and 0.64 (reduction peak), indicating slower reaction kinetics. Notably, despite the carbon layer spacing of HPCV5‐1200 being smaller than HPCV5, the *b* values corresponding to the oxidation and reduction peaks of HPCV5‐1200 increased to 0.77 and 0.84, respectively. This anomalous phenomenon leads us to recognize that the significantly reduced concentration of surface oxygen defects in HPCV5‐1200 played a more critical role in enhancing interfacial charge‐transfer kinetics, ultimately resulting in faster overall Na^+^ storage kinetics.

To further comprehensively understand the kinetic properties of the materials, the capacitive contribution was quantified through calculations by *i* = k_1_ *v* ^1^+k_2_ *v* ^1/2^ formula.^[^ ^45^, ^46^ ^]^ The capacitance control mechanism means that the material has rapid kinetic characteristics, thereby ensuring the excellent rate performance of the carbon materials. As shown in Figures 4d,e and S21 (Supporting Information), HPCV5‐1200 has the highest capacitance ratio (82.2%) at 1.2 mV s^−1^, which is suggestive of that the HPCV5‐1200 owns superior rate performance, compared with other fabricated materials. Figure 4f presents the electrochemical impedance spectroscopy (EIS) spectra of the electrodes, where the mid‐to‐high frequency semicircle corresponds to charge‐transfer resistance and the low‐frequency oblique line represents Na⁺ diffusion resistance.^[^ ^47^ ^]^ After simulations, the charge transfers (R_ct_) were calculated and illustrated as shown in Table S4 (Supporting Information). It can be seen that HPCV5, HPCV5‐1200, and HPCV5‐1400 exhibit more excellent charge transfers than that of HPC, revealing that the carbon coating can enhance the overall conductivity of HPCV materials. Furthermore, the ion diffusion resistances of all electrodes can be determined by the Warburg coefficient (σ) in Figure 4g. It is considerable that a higher σ value indicates poorer ion diffusion performance.^[^ ^48^ ^]^ After calculations, the σ values of HPC, HPCV5, HPCV5‐1200, and HPCV5‐1400 are 250.0, 426.8, 218.0, and 244.0 Ω s^−1/2^, respectively, indicating that HPCV5‐1200 possesses the best ion transport performance.

As shown in Figure 4h, galvanostatic intermittent titration technique (GITT) is used to evaluate the apparent diffusion coefficient of Na^+^ (D_Na+_) of electrodes with a pulse current at 20 mA g^−1^ for 0.5 h between the rest interval for 2 h.^[^ ^15^, ^41^ ^]^ The D_Na+_ calculated from the sodiation and desodiation processes is presented in Figure 4i. In the sodiation process, the D_Na+_ maintains the high values from 1.2 to 0.6 V, indicating a rapid surface adsorption process.^[^ ^49^ ^]^ As the discharge process proceeds, Na^+^ exhibits a characteristic U‐shaped variation (initial decrease followed by an increase) ≈0.6 and 0.1 V (Figure 4h), corresponding to the intercalation and pore‐filling mechanisms of Na^+^ storage, respectively.^[^ ^50^ ^]^ This indicates that sodium ions are initially adsorbed on the material surface, subsequently intercalate into the carbon interlayers, and finally fill the internal nanopores. Nonetheless, both in the slope and plateau region, HPCV5‐1200 exhibits a higher D_Na+_ than the others. This result demonstrates that the defect repair strategy through post‐heat treatment successfully optimizes the oxygen defect concentration within the materials, establishing the unobstructed pathways for efficient Na^+^ diffusion.

**Figure 5:**
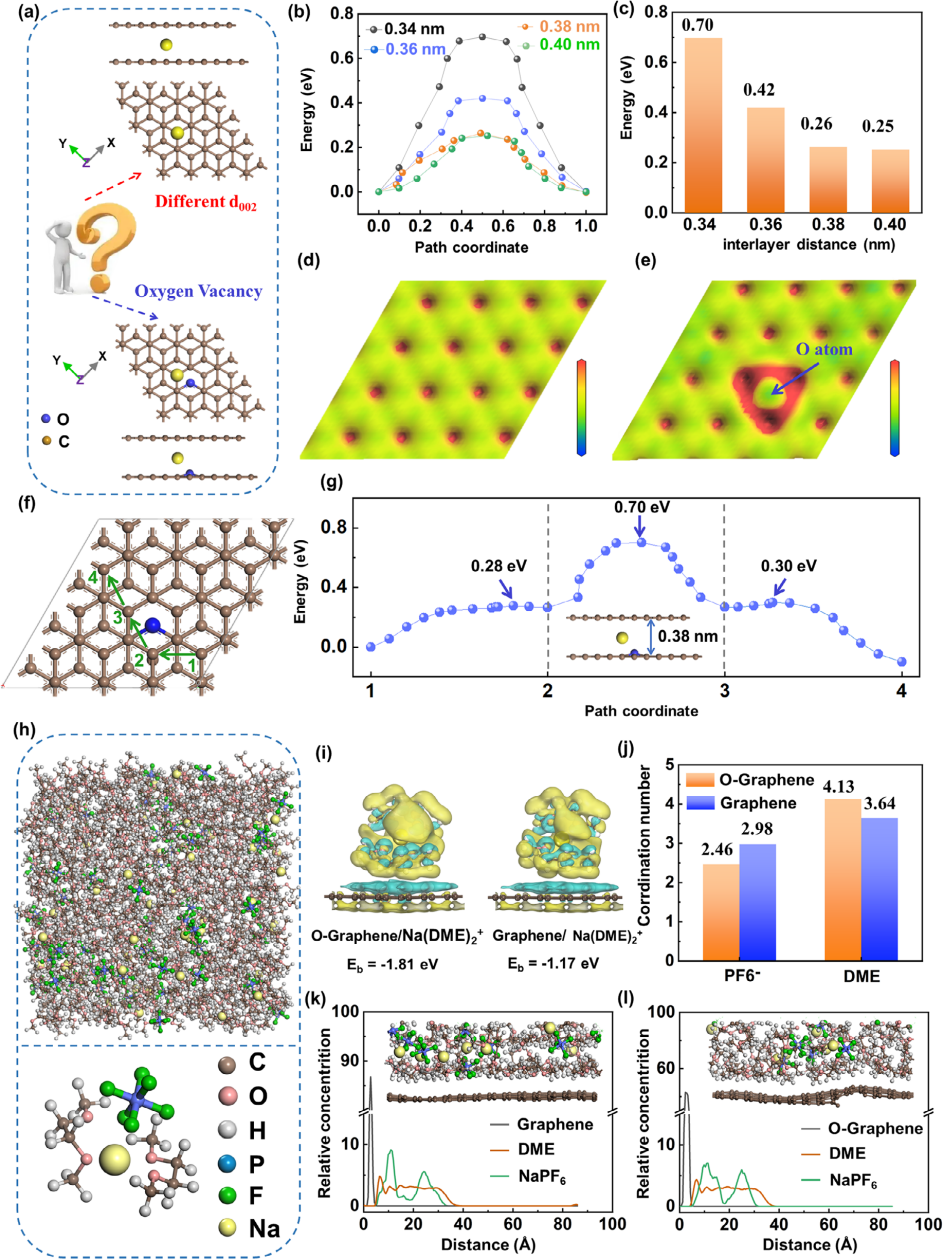
a) Schematic of graphitic layer with specific interlayer distance or oxygen vacancy. b) Diffusion barrier energies of different models. c) The max diffusion barrier energies of different models. Electrostatic potential maps of d) perfect graphene and e) oxygen vacancy graphene. f) Schematic of the Na^+^ migration pathways (1‐2‐3‐4). g) Diffusion barrier energies of Na^+^ migration pathways (1‐2‐3‐4). h) Snapshot of NaPF_6_ in 1 M DME for MD simulation. i) Illustrative charge density difference plots between electrolytic components and carbon electrodes. j) Coordination number for different carbon electrodes. The concentration profile for electrolytic components from MD simulation for k) graphene and l) O‐graphene.

To reveal the deep correlation between the physical parameters and electrochemical performance of HCs, first‐principles calculations were employed to investigate the Na^+^ diffusion behavior in the carbon layers. Representative bilayer graphene models with different interlayer distances (0.34–0.40 nm) were described as shown in **Figures** **5**a and S25 (Supporting Information). As shown in Figure 5b,c, the migration barriers (eV) of Na^+^ in bilayer graphene exhibit a pronounced non‐linear decreasing trend when the interlayer spacing gradually expands from 0.34 to 0.40 nm. The migration barrier of 0.70 eV suggests that the interlayer spacing of 0.34 nm is inadequate for Na^+^ transport, leading to significant electrochemical polarization and consequently impairing the electrochemical performance of the electrode. With further expansion of the interlayer spacing, the migration barriers significantly decrease to 0.26 eV (0.38 nm) and 0.25 eV (0.40 nm), respectively. These highly comparable values indicate that expanding the carbon layer spacing to 0.38 nm enables the rapid intercalation/extraction of Na^+^ within the carbon layers. However, further increasing the carbon layer space to 0.4 nm has a very limited effect on the improvement of Na^+^ diffusion kinetics.

XPS and FTIR analyses revealed that more C─O─C structural defects exist within the materials, which leads us to construct a 0.38 nm interlayer spacing model incorporating C─O─C oxygen defects (Figure S26, Supporting Information). Based on the model in Figure S27 (Supporting Information), Figure 5d,e visualize the electrostatic potentials of pristine graphene and C─O─C oxygen‐defective graphene (O‐graphene). Green regions represent negative potentials (electron‐donating groups), while red regions represent positive potentials.^[^ ^51^, ^52^ ^]^ The results reveal that introducing oxygen defects disrupts the homogeneous electron distribution within the original hexagonal graphene network, thereby inducing the rearrangements of electrons in the carbon skeletons. The strongly electronegative oxygen atoms induce a positive potential (red areas) in adjacent regions (Figure 5e), producing a local charge repulsion effect that significantly hinders the migration of positively charged Na^+^ ions. The calculations confirm that the migration barriers of Na^+^ in bilayer graphene with C─O─C defect are as high as 0.70 eV, which is much higher than Na^+^ in the perfect bilayer graphene model with the same layer spacing (0.26 eV) (Figure 5g). Therefore, the rational design of oxygen concentrations in carbon materials, balancing the impact of carbon layer spacing and oxygen defects on Na^+^ diffusion kinetics, is crucial for achieving the high Na^+^ storage performance. The further calculations regarding the effects of carboxyl and carbonyl substituent groups on Na^+^ adsorption and diffusion are presented in Figures S28 and S29 (Supporting Information).

It is acknowledged that the SEI is also one of the decisive factors for the reversibility, capability, and cyclability of carbon materials. To deeply analyze the formation of the SEI film at electrode/electrolyte interfaces, the molecular dynamics (MD) simulations of realistic electrolyte environments were utilized by computational analyses. It is found that the Na^+^ tends to bind with 1,2‐dimethoxyethane (DME) in the form of Na(DME)^2+^, which couples with PF_6_^−^ in the electrolyte (1 m NaPF_6_ in DME) (Figure 5h). The reactivity between Na(DME)_2_^+^ and carbon electrodes is also quantified by the DFT calculation. Figure 5i shows the charge‐transfer at different electrode and electrolyte interfaces. Higher charge accumulation and depletion regions are observed in O‐Graphene/Na(DME)_2_^+^, which means that a large charge‐transfers occur between O‐Graphene and DME. Compared to pristine graphene, the larger binding energy of Na(DME)_2_^+^ on O‐graphene indicates that oxygen defects (C─O─C) facilitate Na(DME)_2_^+^ adsorption and subsequent electrolytic reduction, thereby increasing the proportion of organic components in the SEI.^[^ ^53^ ^]^ Figures 5j and S32 (Supporting Information) present the coordination number (CN) analysis around Na^+^ and the corresponding radial distribution functions of Na^+^‐F (Na^+^‐PF_6_^‐^) and Na^+^‐O (Na^+^‐DME). The O‐graphene interface exhibits a CN (4.13) for DME and a CN (2.46) for PF_6_^−^ anion. The graphene interface (deoxygenated interfaces) demonstrates the reduced CN of (3.64, Na^+^‐O) and increased CN of (2.98, Na^+^‐F), which indicates greater PF_6_^−^ anion participation in the solvation sheath, leading to generate the inorganic‐rich SEI. The weak solvating effect at the electrolyte/carbon interface is further revealed in Figure 5k,l. In the O‐graphene/electrolyte system, the position of DME solvent is closer to the carbon surface than that of PF_6_^−^ anions, where the PF_6_^−^ anionic group is excluded from the interface, resulting in a higher organic content in the resulting SEI. Conversely, the graphene/electrolyte system effectively suppresses this strongly solvating effect, enabling more PF_6_^−^ anions to access to the interface and facilitating inorganic‐rich SEI formation. Theoretical calculations provide in‐depth insights into the role of oxygen defects in regulating Na^+^ diffusion kinetics from both the physical structure and surface chemical property of carbon materials, and also elucidate the fundamental reason for the enhanced kinetics of HPCV5‐1200, after the deoxygenation via post‐heat treatment of HPCV5.

**Figure 6:**
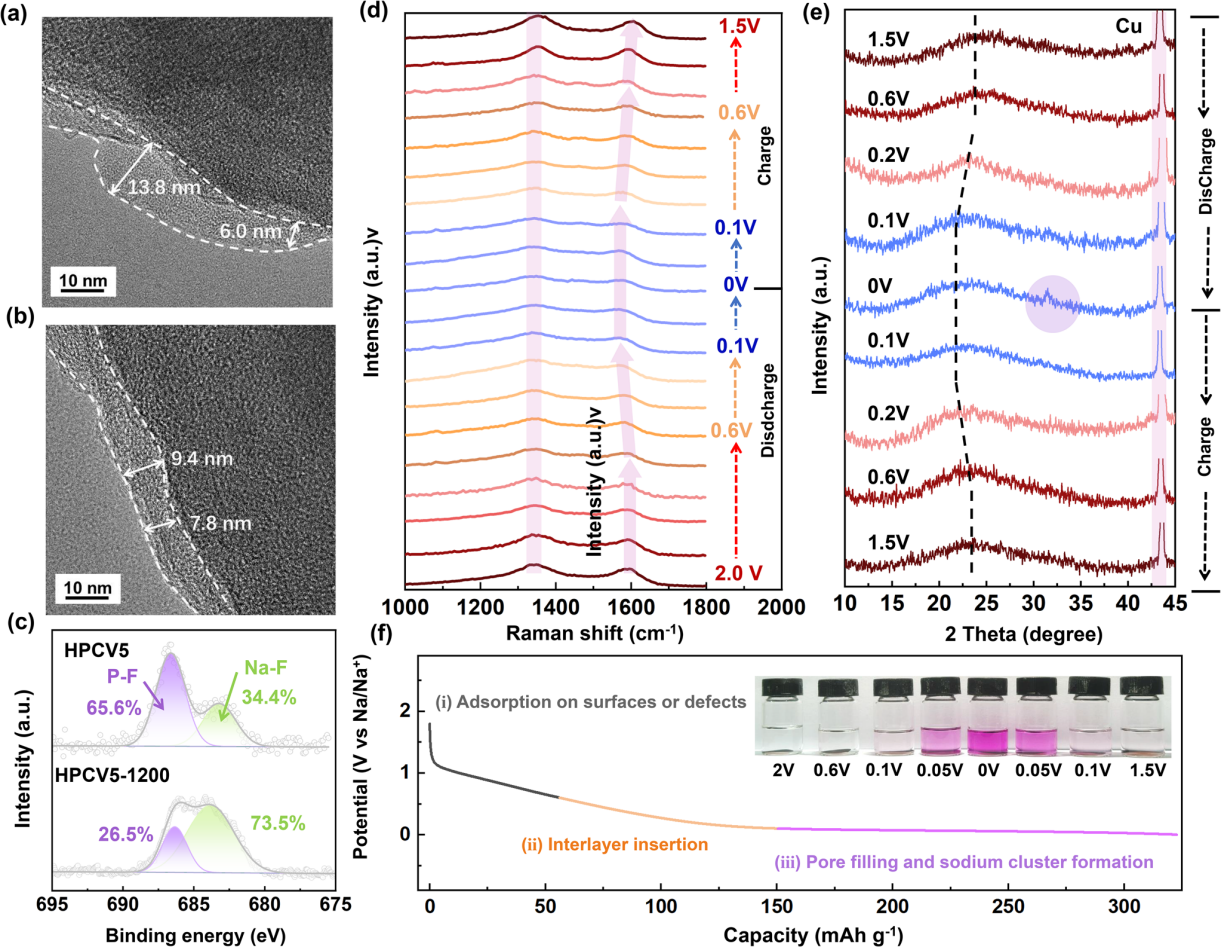
TEM images of a) HPCV5 and b) HPCV5‐1200 after five cycles. c) XPS spectra of F 1s on HPCV5 and b) HPCV5‐1200 after five cycles. d) In situ Raman spectra and e) ex‐XRD patterns of the HPCV5‐1200 electrode. f) Schematic diagram of the Na^+^ storage stages of HCs. Insets show the corresponding color change of ethanol containing 1% phenolphthalein, after reaction with the HPCV5‐1200 electrode at different potentials.

The SEI formation and growth were characterized by TEM measurements using the HCs/Na half‐cells which are performed the discharge–charge three cycles. As shown in **Figure** **6**a,b, the anode of HPCV5 has a rough SEI, and its thickness distribution is uneven. In contrast, HPCV5‐1200 features a smoother and more uniform SEI layer, which reflects that the deoxidized surface can effectively suppress the side reactions of interfacial electrolysis. The XPS results further reveal SEI compositional changes (Figures 6c; S33, Supporting Information). The two C 1s peaks at 286.04 eV (C─O), 286.8 eV (O─C═O) belong to the RCH_2_ONa, and 288.3 eV (CO_3_) can be ascribed to Na_2_CO_3_. The two compounds are produced by the combination of Na^+^ and the decomposition products of DME.^[^ ^21^, ^53^, ^54^, ^55^ ^]^ The higher ratio of C─O and O─C═O in HPCV5 represents higher RCH_2_ONa contents, further demonstrating that oxygen defects in the surface of carbon coatings promote the accumulation of organic components in the SEI. The intensity of F 1s peak in HPCV5‐1200 confirms the higher NaF content (Figure 6c), which enhances SEI rigidity and stability for reversible Na^+^ storage.^[^ ^53^, ^56^ ^]^ These experimental observations collectively validate the results of the DFT calculations.

The structural evolution of HPCV5‐1200 during the cycling was investigated by using in situ Raman spectroscopy and ex situ XRD. As shown in Figure 6d, during the discharge processes from 2.0 to 0.6 V, the D peak exhibited a gradual widening, however, the peak position (1597 cm^−1^) remained constant. This observation indicates that Na^+^ storage within this voltage range is primarily governed by an adsorption mechanism.^[^ ^57^ ^]^ In the voltage range of 0.6–0.01 V, the G peak exhibits a gradual shift from 1597 to 1570 cm^−1^ toward lower frequencies. This phenomenon is attributed to the Na^+^ intercalation in the carbon layer, causing electrons to occupy the π^*^ antibond orbitals and form Na─C bonds, thereby weakening the C─C bond vibration and causing the redshift of the G peak.^[^ ^28^, ^39^, ^58^ ^]^ In the voltage range of 0.1–0 V, the G peak stops shifting and the intensity of the D peak significantly weakens, indicating that the defects in carbon surface is covered by the Na metal clusters.^[^ ^39^, ^59^ ^]^ During the charging process, it is observed that the Raman spectrum of materials could return to its initial shape, indicating that the Na^+^ deintercalation restored the microcrystalline structure. The ex situ XRD patterns of HPCV5‐1200 at different voltages (Figure 6e) reveal that the (002) peak shows no change during discharges from 1.5 to 0.6 V, further confirming that sodium‐ion storage occurs primarily via a surface adsorption mechanism. When discharging to the 0.1 V, it is obvious that the peak shift to a low angle, which is ascribed to that interlayer spacing is expanded due to the Na^+^ intercalation in the carbon layers. Upon discharging to 0 V, the (002) peak shows no significant shift, while the characteristic peak of metallic sodium appears at 31.3°,^[^ ^49^, ^59^ ^]^ indicating that the Na^+^ storage mechanism in the low‐plateau region is dominated by pore filling. During the charging process, the XRD pattern of the material fully returned to its initial state, indicating that the structural recovery after Na^+^ extraction is reversible, which is consistent with the in situ Raman results. Furthermore, the ex situ XPS measurements are utilized to further investigate the Na^+^ storage mechanism of HPCV5‐1200 (Figure S34, Supporting Information).

The reaction of sodium metal with ethanol produces H_2_ and sodium ethoxide (CH_3_CH_2_ONa),^[^ ^39^ ^]^ turning the phenolphthalein solution to red color. Therefore, this experimental method is further utilized to confirm the formation of metallic sodium. As shown in Figure 6f, the solution color gradually turns purple with the decreasing of discharge voltages, indicating the continuous formation of metallic sodium. Video S1 (Supporting Information) shows the image of placing the HPCV5‐1200 electrode in ethanol phenolphthalein solution when discharging to 0.01 V. It can be observed that a large number of bubbles produced on the surface of the HPCV5‐1200 electrode and the solution color turns dark purple (Video S1, Supporting Information), which further indicates the presence of metallic sodium in the HPCV5‐1200 electrode. On the contrary, the color of the solution gradually becomes lighter, as the charging process proceeds. The video results strongly suggest the results of in situ Raman and ex situ XRD results. The aforementioned analyses indicate that the Na^+^ storage mechanism mainly consists of a three‐step processes: Na^+^ adsorption, Na^+^ intercalation, and finally filling into closed pores in carbon anodes.

## 3 Conclusion

In summary, HPCV materials with high Na^+^ storage performance were prepared through a novel molecular cross‐linking CVD method and subsequent heat treatment processes. The microcrystalline optimization, pore reconstruction, and interface adjustment of fabricated carbons have been reasonably achieved. On this basis, it is found that the positive effect of well‐regulated O defect concentration on improving Na^+^ storage capacity and rate performance was revealed. Overall, the result indicates that molecular cross‐linking suppresses *π–π* stacking in PAHs, creating expanded interlayer spacing and developed pore architecture in CTPC, while the following CVD process facilitates the crucial open‐to‐closed pore transformation. Controlling the post‐heat temperatures, the d‐spacing parameters, and O defect concentrations of the carbon coating layers can be well adjusted, which plays a significant role in enhancing the Na^+^ transfer mobility and the construction of inorganics‐enriched SEI on carbon anodes. After conducting the electrochemical evaluations, the optimized HPCV5‐1200 electrode demonstrates a specific capacity of 320.2 mAh g^−1^ after 300 cycles at a current density of 0.2 A g^−1^, along with excellent high‐rate performance, as evidenced by specific capacities of 302.8 mAh g^−1^ at 0.1 A g^−1^, 229.3 mAh g^−1^ at 2.0 A g^−1^, 187.6 mAh g^−1^ at 5.0 A g^−1^, and 112.5 mAh g^−1^ at 10.0 A g^−1^. Furthermore, detailed DFT calculations show that the Na^+^ transfer kinetics of HCs anodes is not only controlled by the traditional understanding of layer spacing, but also seriously affected by the local electronic environment of the carbon framework. The MD simulation reveals that carbon coatings with low oxygen content attract more PF_6_^−^ anions to reach the interface of electrodes, generating an inorganic‐enriched SEI. Through synergistic defect engineering and structural optimization, the kinetic limitations of HCs anodes can be overcome, providing a new reference for developing SIBs with the high energy density.

## Conflict of Interest

The authors declare no conflict of interest.

## Supporting information



Supporting Information

Supplementary Video 1

## Data Availability

Research data are not shared.
