# Supplementary material for: Dual Regulation on Structure‐Interface Enables Coal‐Tar‐Pitch‐Based Hard Carbon Anodes with High Rate and Storage Performance for Sodium Ion Batteries
Source: Adv Sci (Weinh). 2025 Oct 5;12(48):e15146. doi: 10.1002/advs.202515146 (PMC12752629; doi:10.1002/advs.202515146)
Supplement: Supplementary file 1 — Supporting Information [file ADVS-12-e15146-s002.pdf]

Supporting Information

Dual Regulation on Structure-Interface Enables Coal-Tar-Pitch Based Hard Carbon Anodes with High Rate and Storage Performance for Sodium Ion Batteries

Xinmeng Xu[a], Kun Wang[a,*] Beibei Han[c], Jianke Li[a], Baigang An[a], Chengguo Sun[a], Guiying Xu[a,*] Zewei Li[d], Wenwu Zhang[e] , Zhenbo Wang[b], Weimin Zhou[a,*]

[a] Key Laboratory of Energy Materials and Electrochemistry Research Liaoning Province, University of Science and Technology Liaoning, No. 189, Qianshan Middle Road, Lishan District, Anshan City, Liaoning Province, Anshan 114051, China.
[b] State Key Laboratory of Space Power-Sources, MIIT Key Laboratory of Critical Materials, Technology for New Energy Conversion and Storage, MOE Engineering Research Center for Electrochemical Energy Storage and Carbon Neutrality in Cold Regions, School of Chemistry and Chemical Engineering, Harbin Institute of Technology, Harbin 150001, China.
[c] Key Laboratory of Advanced Fuel Cells and Electrolyzers Technology of Zhejiang Province, Ningbo Institute of Materials Technology and Engineering, Chinese Academy of Sciences, Ningbo, No. 1219 Zhongguan West Road, Zhejiang 315201, China.
[d] Weida New Material Technology Co., Ltd., Jixi 158100, China.
[e] Haicheng Shenhe Technology Co., Ltd, Haicheng 114213, PR China.

Corresponding E-mail: wk172860@ustl.edu.cn; xuguiying751107@ustl.edu.cn; aszhou@ustl.edu.cn

Experimental section

Materials

Coal-tar-pitches (CTPs, soften point 95 ℃) was provided by Angang Steel Group Limited, Trichloromethane (≥ 99.0%), aluminum chloride (≥ 99.0%), hydrochloric acid (37.5 wt%) and anhydrous ethanol (99.5%) was bought from Sinopharm Chemical Reagent Co., Ltd.

Preparation of HPC

CTPs (1g) were dissolved in the $CHCl_3$ (30 mL). After the obtained solution was conducted the ultrasonic treatment for 30 min, the anhydrous aluminum chloride $AlCl_3$ (3 g) was added and reacted in a water bath at 58 ℃ for 24 h. After the reaction, the product was washed with 3 mol/L hydrochloric acid and anhydrous ethanol, and then the Hyper-crosslinked coal tar pitches (HCL-CTPs) was obtained. The HCL-CTPs were placed in a tube furnace, in which the temperature was increased to 1200 ℃ for 2h with a rate of 5 ℃/min under an argon atmosphere. After cooling to room temperature, the HCL-CTPs-based hard carbon (HPC) was collected.

Preparations of HPCV materials

CTPs and HPC were placed in the tube furnace in the mass ratio of 3:1, 5:1, and 7:1 respectively, along the flow direction of the inert gas. The tube furnace temperature was raised to 1000 ℃ for 2h with a heating rate of 5 ℃/min. After cooling down to room temperature, the obtained samples were named as HPCV3, HPCV5 and HPCV7, respectively. Among them, the HPCV5 was further performed the carbonization at temperature 1200 ℃ and 1400 ℃ for 2h, respectively. The obtained materials were named as HPCV5-1200 and HPCV5-1400, respectively. The preparation procedure is illustrated as shown in Figure S1.

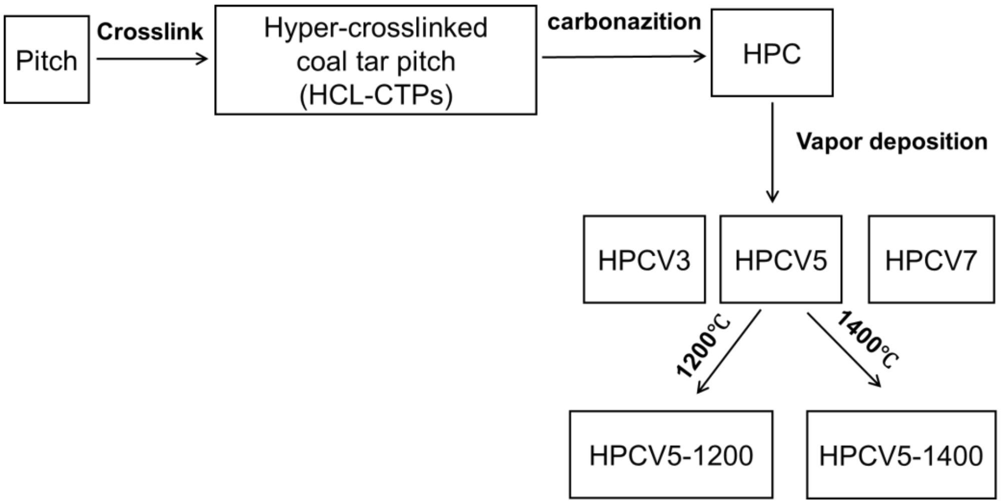

Figure S1. The synthesis process of HPC and HPCV materials.

Characterization

The X-ray diffraction (XRD) patterns were measured with an X'pert Powder instrument from PANalytical at 40.0 kV and 40 mA with Cu-Kα radiation. The results of X-ray photoelectron spectroscopy (XPS) were verified by a K-Alpha instrument using an Al-Kα source (12 kV) from Thermo Fisher Scientific, USA. The Raman spectra were recorded on the HR 800 laser Raman spectrometer of Horiba Jobin Yvon company, France. In-situ Raman measurements were conducted through a customized cell device. Among them, the observation window of the in-situ Raman system was quartz. Thermal gravimetric analyzer (TGA) measurements were carried out by the NETZSCH TG209F3 (Germany) with a heating rate of 10 ℃/min. Nitrogen adsorption and desorption isotherms were measured by an autosorb-iQ surface analyzer which was purchased from Quantachrome Instruments, USA. The specific surface area was evaluated, according to the Brunauer-Emmett-Teller (BET) method. Based on a DFT model, the size distributions were evaluated. TEM measurements were performed on the HF-3300 system (Hitachi Co. Ltd., Tokyo, Japan). Ture density test was carried out by an AccuPyc II 1340 analyzer using Helium as analysis gas. Cyclic voltammetry (CV) were tested by CHI660E electrochemical workstation (ChenHua,

Shanghai, China). The structures of the HCl-CTPs were further characterized by using a ^{13}C cross-polarization magic angle spinning nuclear magnetic resonance spectrometer (Bruker Avance Neo 400WB , Germany).

Electrochemical Measurements

The electrode was prepared from a mixture of active material, conductive carbon black, and CMC at a weight ratio of 90:5:5. This mixture was dispersed in purified water to form a slurry, which was uniformly coated onto a copper foil current collector. After vacuum drying at 120 °C performed for 12 h, the copper foil was punched into a circular electrode with a diameter of 10 mm. The mass loading of active materials was approximately controlled as ~1 mg cm^{-2}. The CR2032-type coin cells were obtained in glove box using sodium foil as counter electrodes, the glass fiber (Whatman, GF/D) as separators, and the electrolyte (1M $NaPF_6$ in 1,2-dimethoxyethane). The charge-discharge test was performed on the LAND CT3001A. The test voltage window is 0-3V at room temperature.

DFT calculations

The DMol3 quantum mechanics program based on DFT is used for geometry optimization.[[1],[2]] A 4×4×2 super cell and a 20 Å vacuum space along the z-axis for graphitic layers (AB stacking) was adopted. Generalized gradient approximation (GGA) with Perdew-Burke-Ernzerhof (PBE) was chosen as exchange-correlation function. A 3×3×1 k-point mesh was adopted by the Monkhrost-Pack technique to sample the Brillouin zone. The effects of dispersion interaction are considered utilizing the empirical correction scheme of Grimme (DFT-D2) as implemented.[[3]] The nudged elastic band (NEB) method was used to carefully study the influence of different microstructures on the Na^+ diffusion pathway and minimum diffusion energy barrier of different

models.[[4]] The calculation for differential charge density is from Cambridge Sequential Total Energy Package (CASTEP) based on DFT. MD simulations were performed based on the Forcite module with COMPASS II force filed. A 100 ps simulation was run by using the NPT ensemble before 200 ps to reach equilibrium using the NVT ensemble at 298 K. For the establishment of the electrolyte/carbon interface, 50 Å of vacuum was added to form the slab model.[[5]]

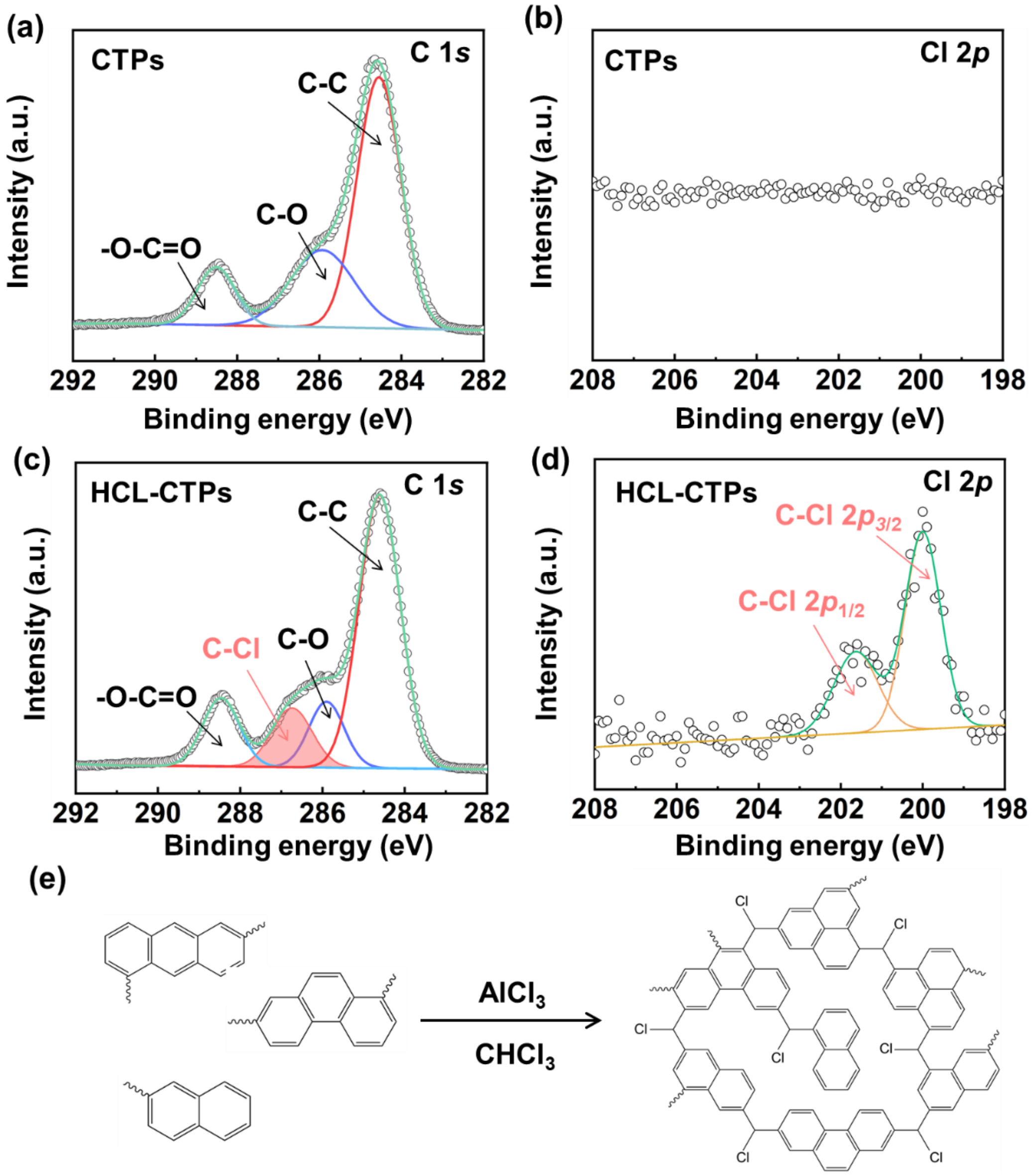

Figure S2. The high-resolution C 1s spectra of a) coal tar pitches (CTPs) and c) hyper-crosslinked coal tar pitches (HCL-CTPs). The high-resolution Cl 2p spectra of b) CTPs and d) HCL-CTPs. e) Schematic diagram of the preparation of HCL-CTPs.

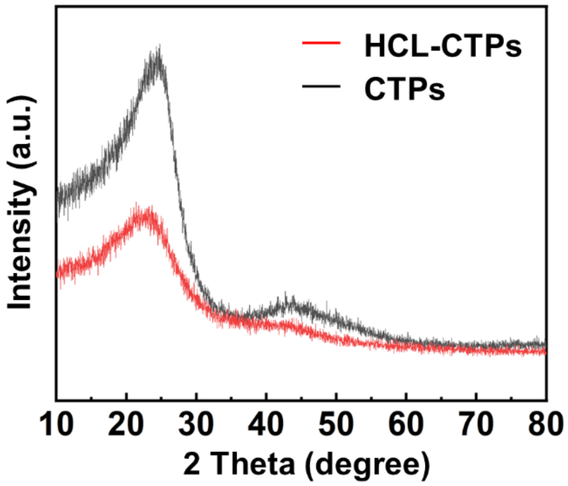

Figure S3. XRD pattern of CTPs and HCL-CTPs.

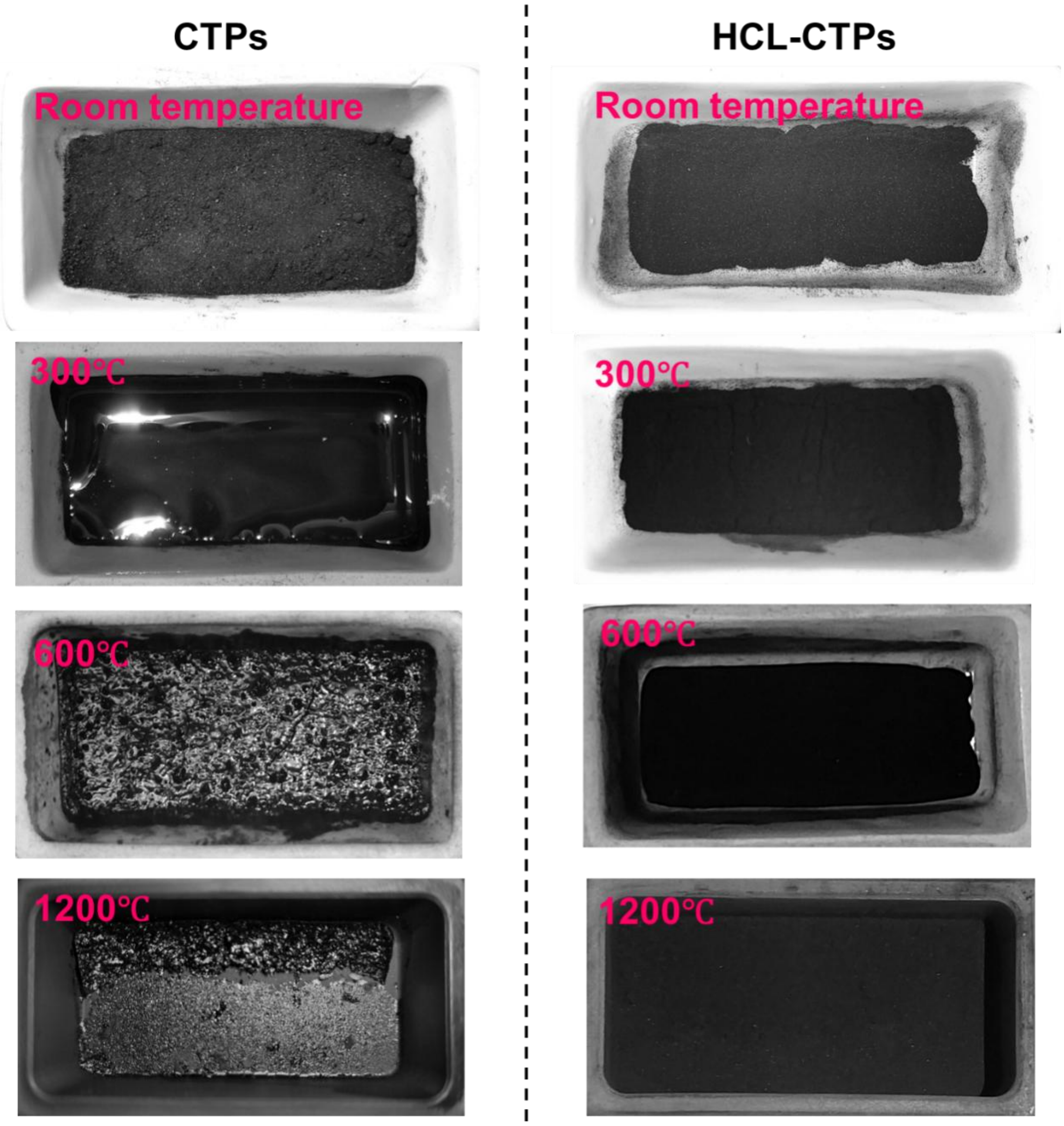

Figure S4. Optical photographs of CTP and HCL-CTPs at different temperatures.

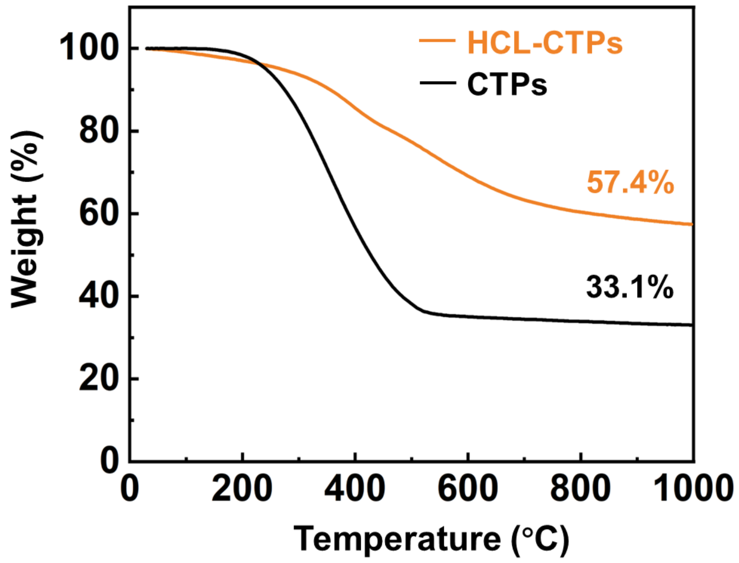

Figure S5. TGA curves of CTPs and HCL-CTPs.

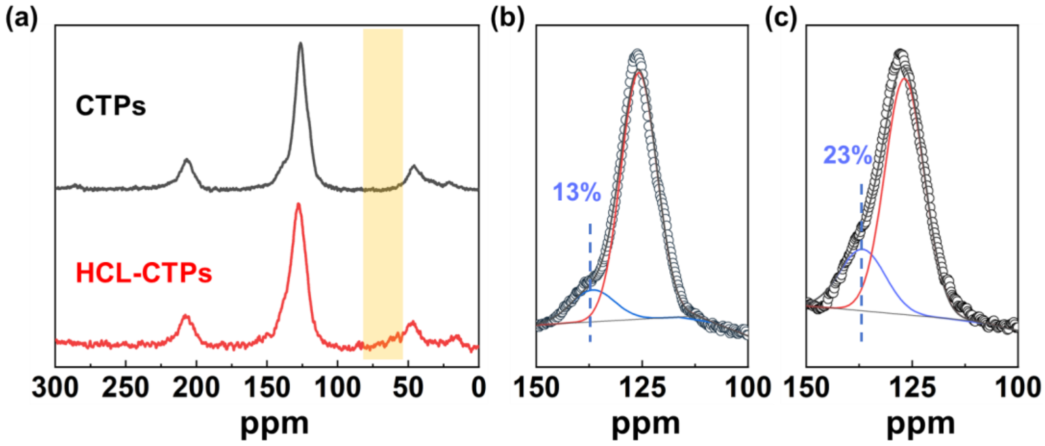

Figure S6. a) Solid state ^{13}C cross-polarization nuclear magic-angle spinning (CP/MAS) NMR spectra of coal-tar-pitches (CTPs) and hyper-crosslinked coal-tar-pitches (HCL-CTPs) at 0-300 ppm. Solid state ^{13}CCP/MAS NMR spectra of b) CTPs and c) HCL-CTPs at 100-150 ppm.

The structures about crosslinking of PAHs were further investigated by using a ^{13}C cross-polarization magic angle spinning nuclear magnetic resonance (CP/MAS NMR) spectrometer) (Bruker Avance Neo 400WB). As shown in Figure S6, the broad peak observed around 58-70 ppm in the HCL-CTPs (hyper-crosslinked coal-tar-pitches) spectrum is attributed to methylene carbons that are linked with the heteroatoms.[6] Additionally, the peak at 137 ppm represents the substituted aromatic carbon.[6] Following Friedel-Crafts alkylation crosslinking, the peak intensity at 137 ppm in HCL-CTPs increased from 13% to 23%, indicating substitution of aromatic rings. This data further confirms the successful formation of the crosslinked network in coal-tar-pitches.

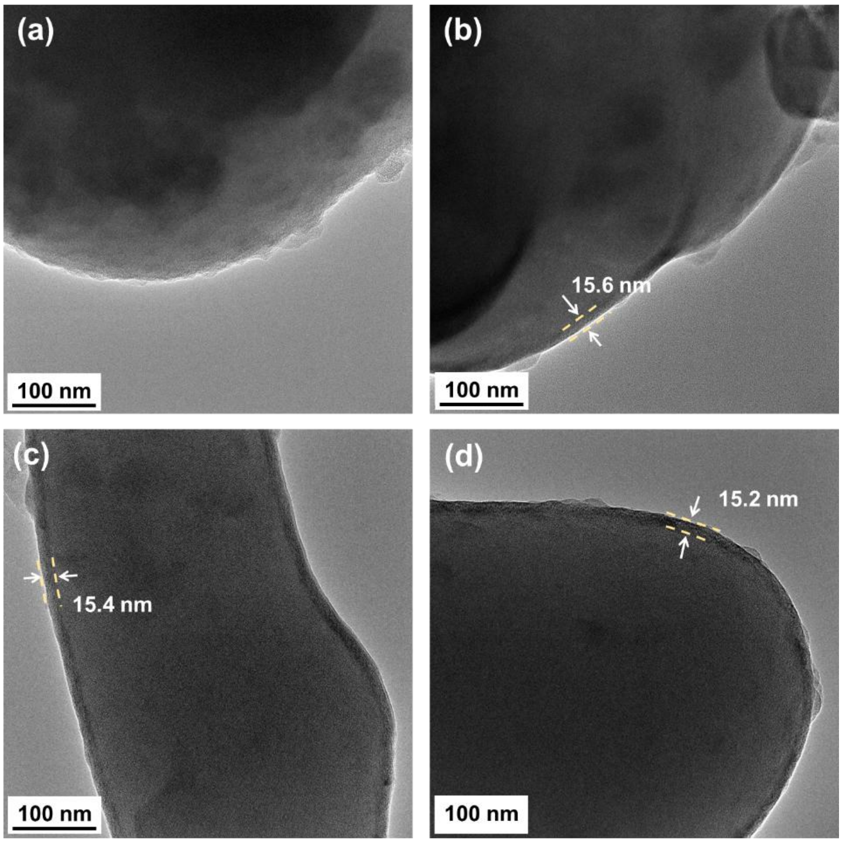

Figure S7. TEM images of a) HPC, b) HPCV5, c) HPCV5-1200 and d) HPCV5-1400.

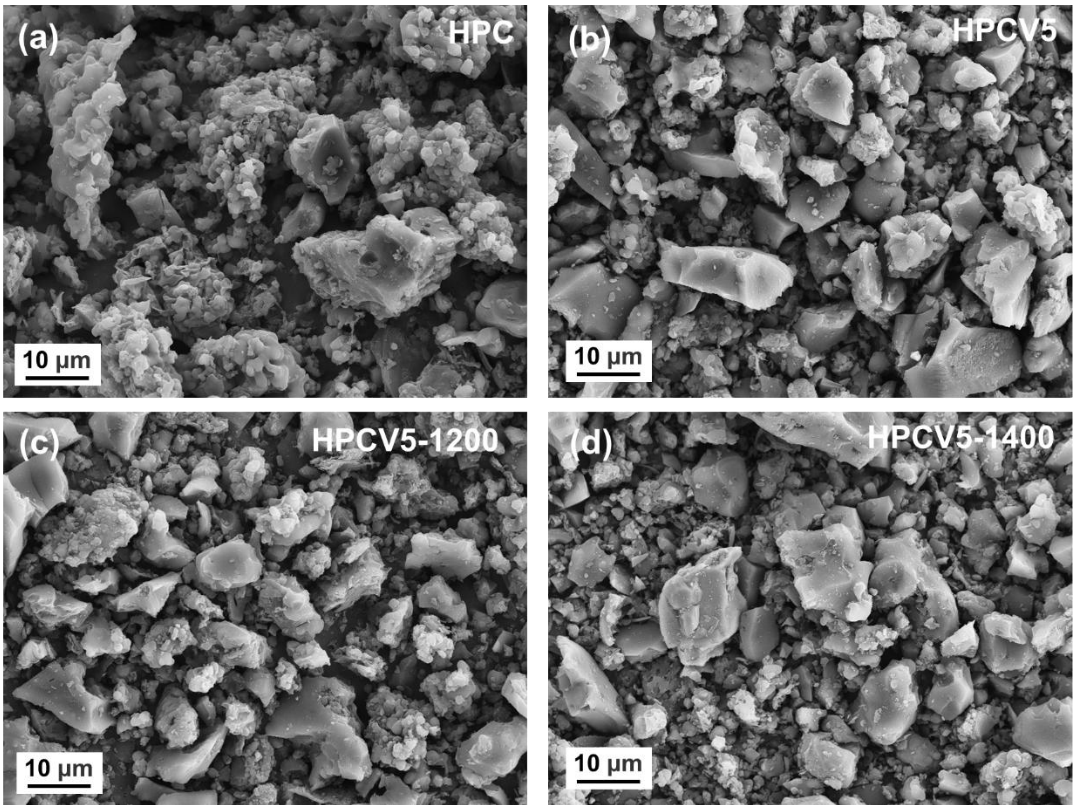

Figure S8. SEM images of a) HPC, b) HPCV5, c) HPCV5-1200 and d) HPCV5-1400.

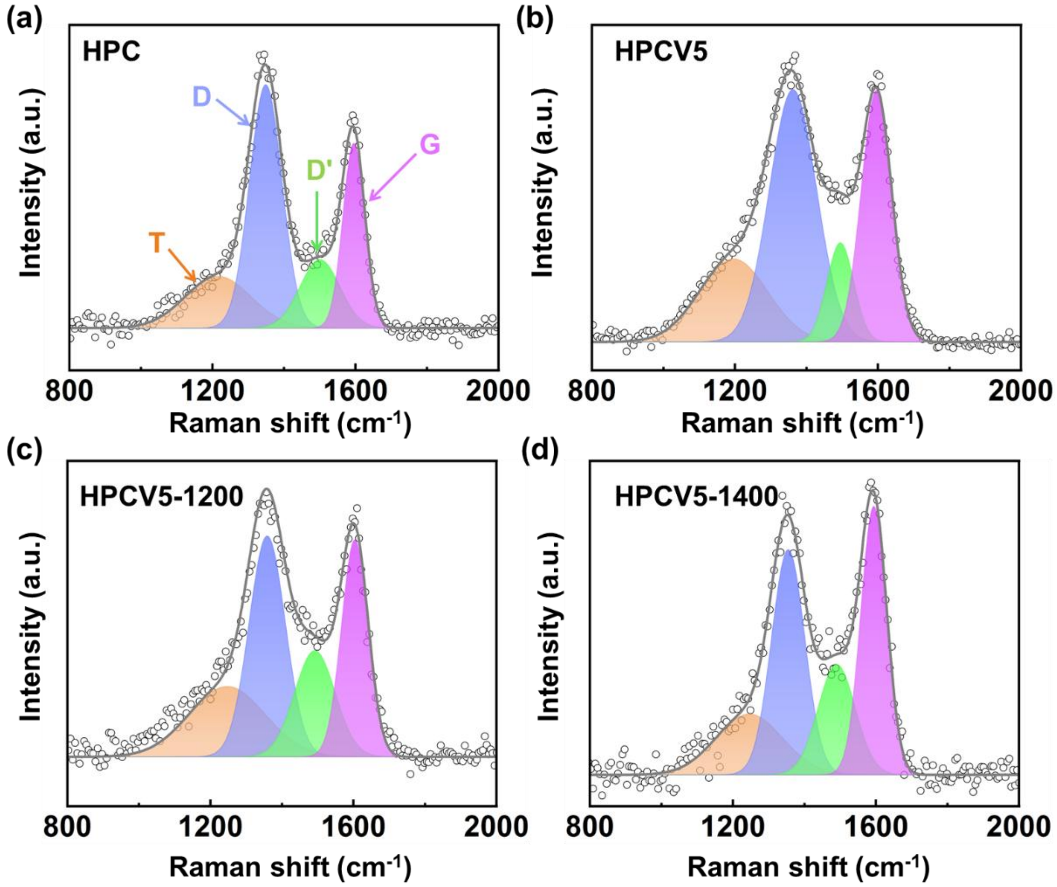

Figure S9. Raman spectra of a) HPC, b) HPCV5, c) HPCV5-1200 and d) HPCV5-1400.

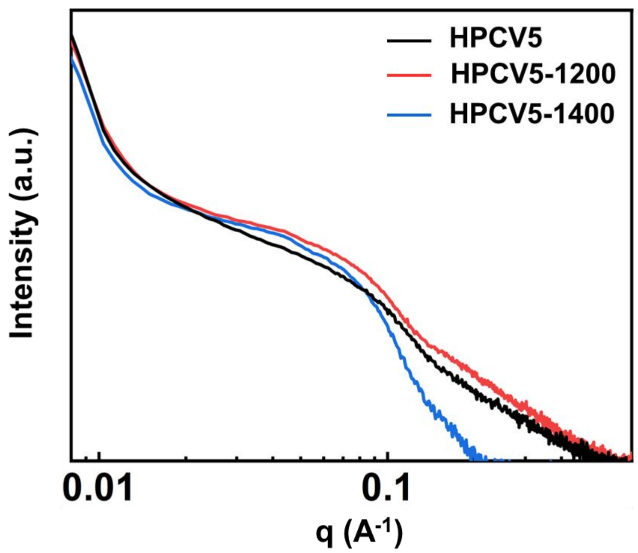

Figure S10. SAXS curves of HPCV5, HPCV5-1200 and HPCV5-1400.

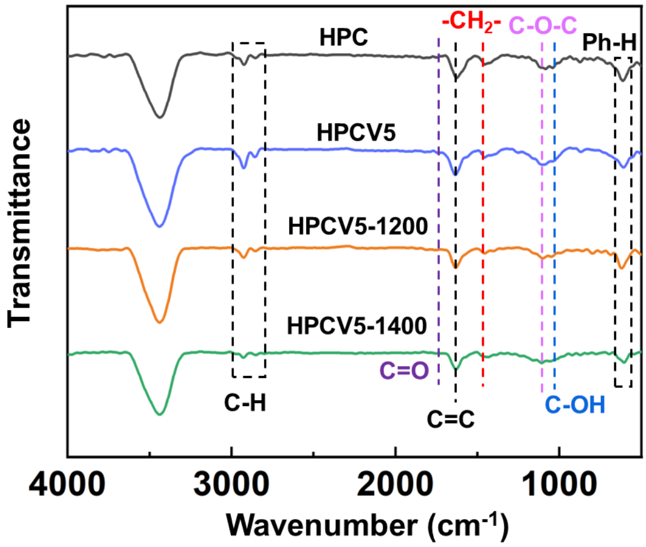

Figure S11. FT-IR spectra of HPC, HPCV5, HPCV5-1200 and HPCV5-1400.

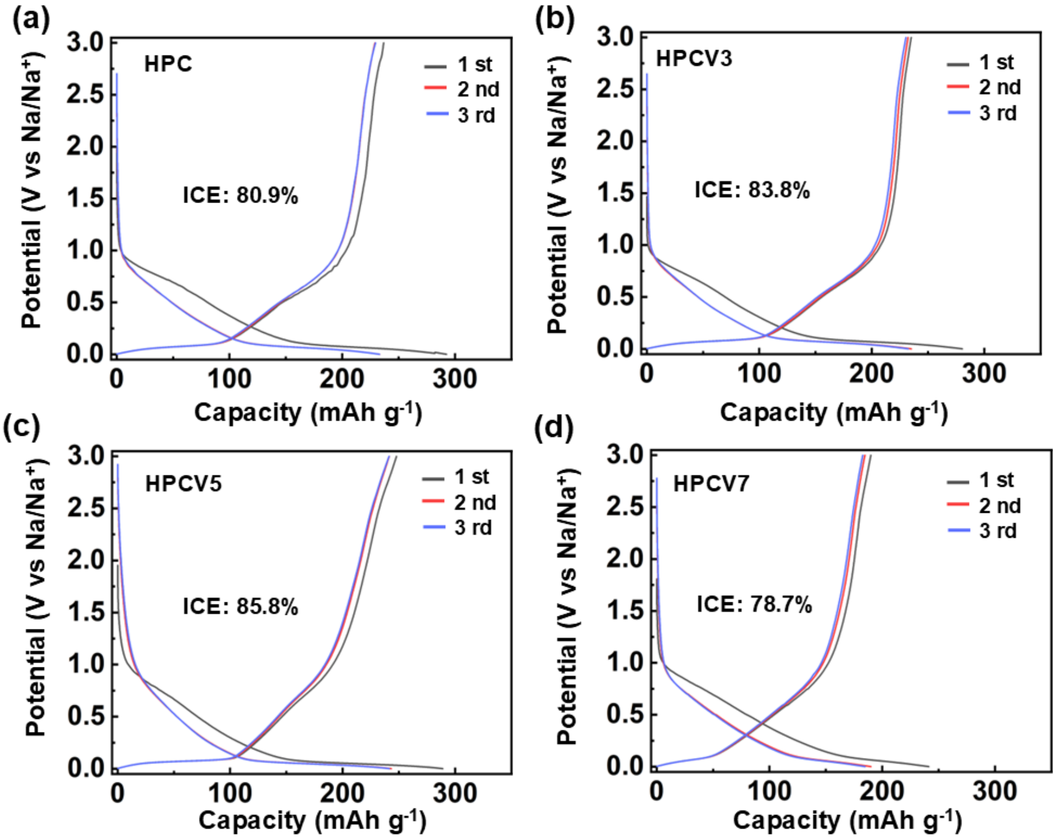

Figure S12. The discharge-charge profiles of a) HPC, b) HPCV3, c) HPCV5 and d) HPCV7.

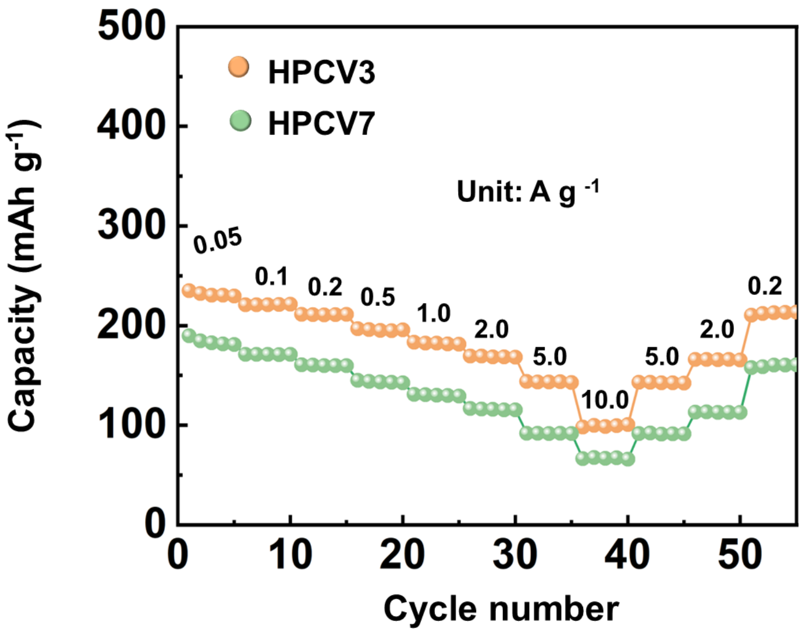

Figure S13. Rate performance at different current densities.

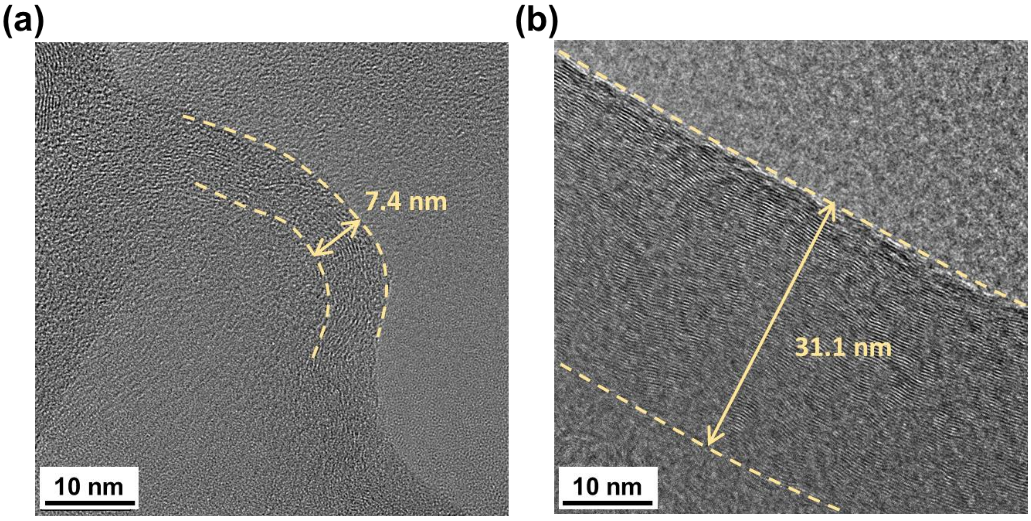

Figure S14. HRTEM images of a) HPCV3 and b) HPCV7.

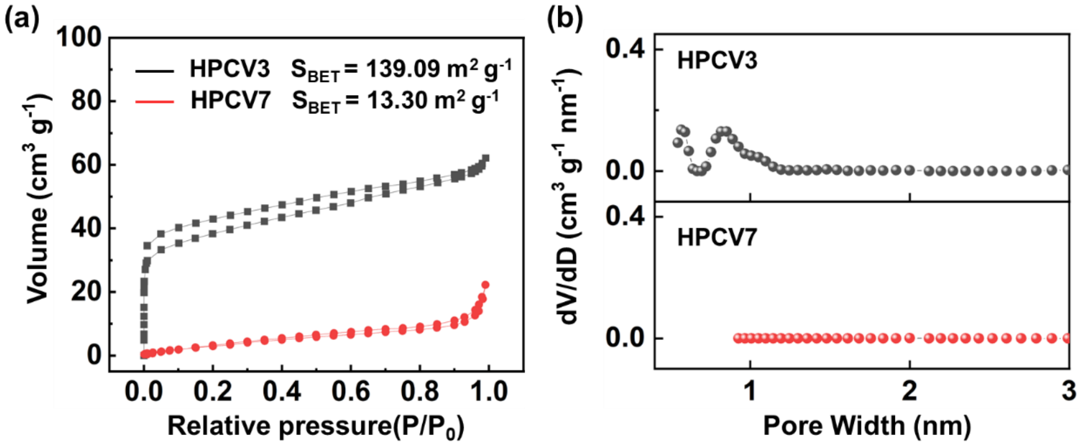

Figure S15. a) N_2 adsorption-desorption isothermal curves of HPCV3 and HPCV7. b) The corresponding pore size distributions of HPCV3 and HPCV7.

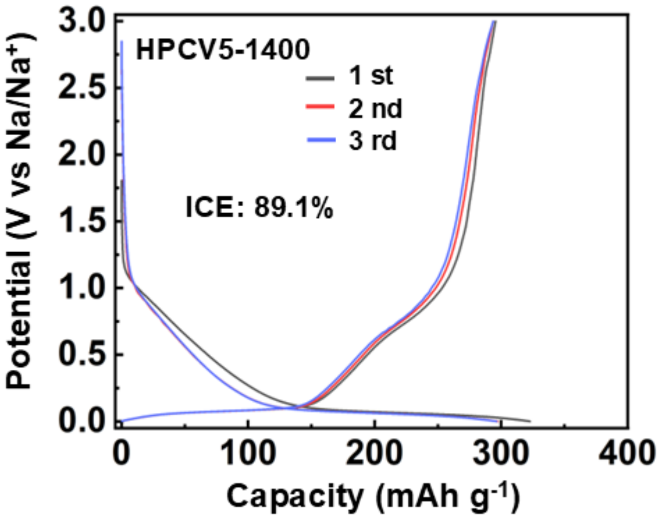

Figure S16. The discharge-charge profiles of HPCV5-1400.

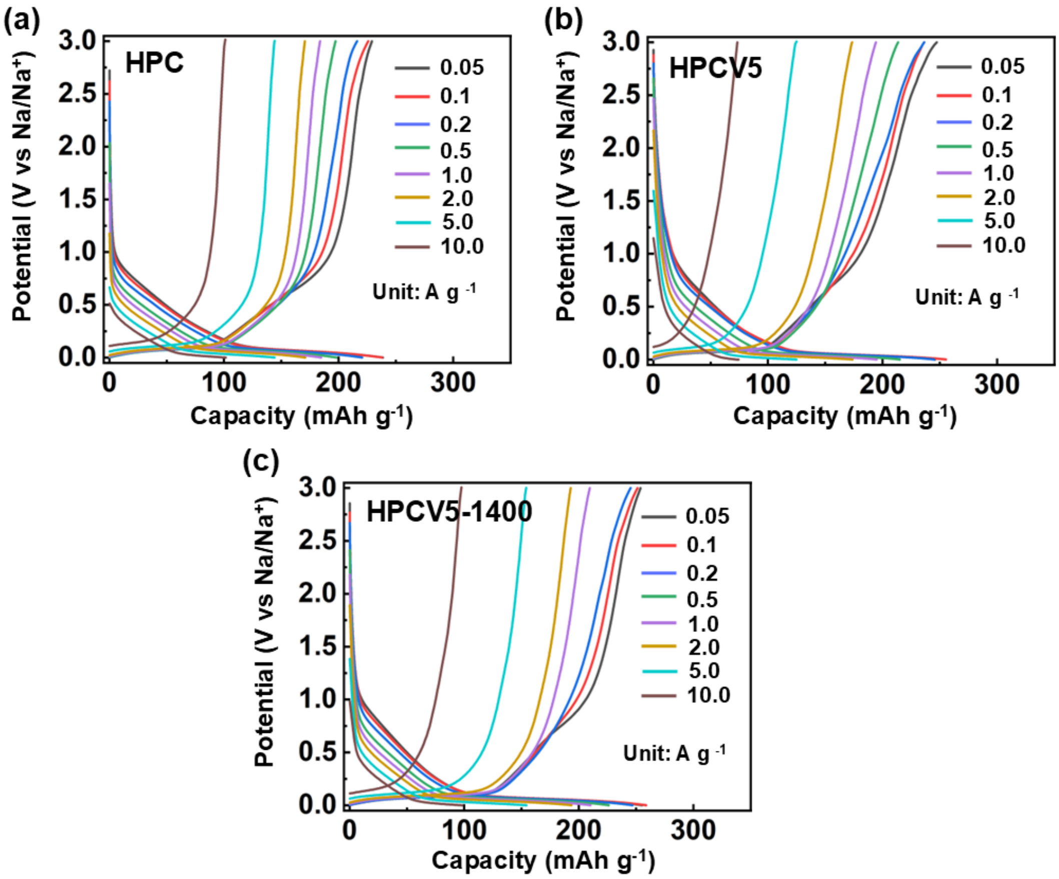

Figure S17. Galvanostatic charge-discharge curve of a) HPC, b) HPCV5 and c) HPCV5-1400 at different current densities.

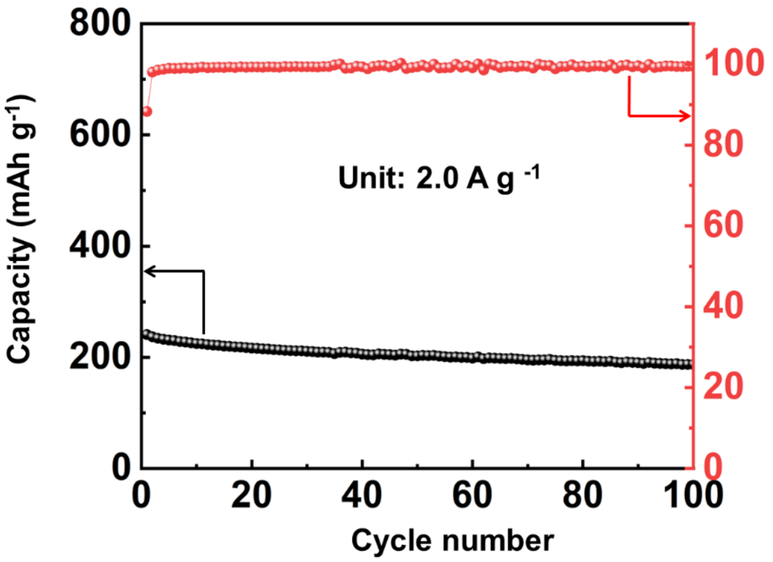

Figure S18. Cycle performance of full cell at 2 A g^{-1}.

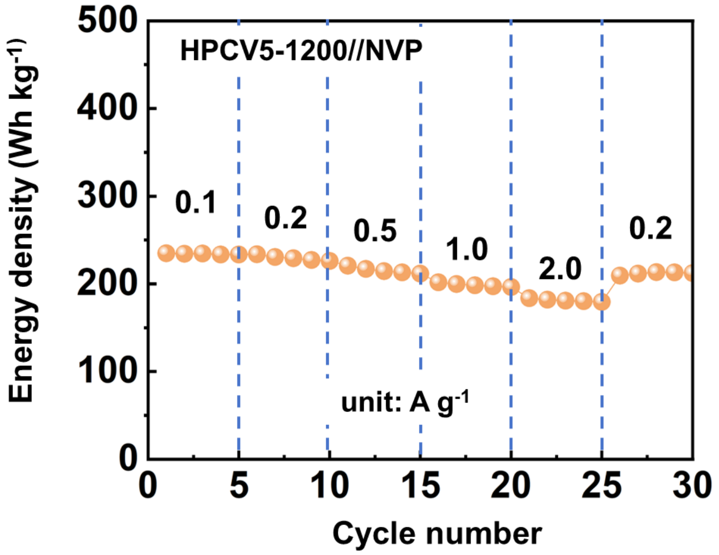

Figure S19. Rate performance at different current densities of full cell (HPCV5-1200//NVP).

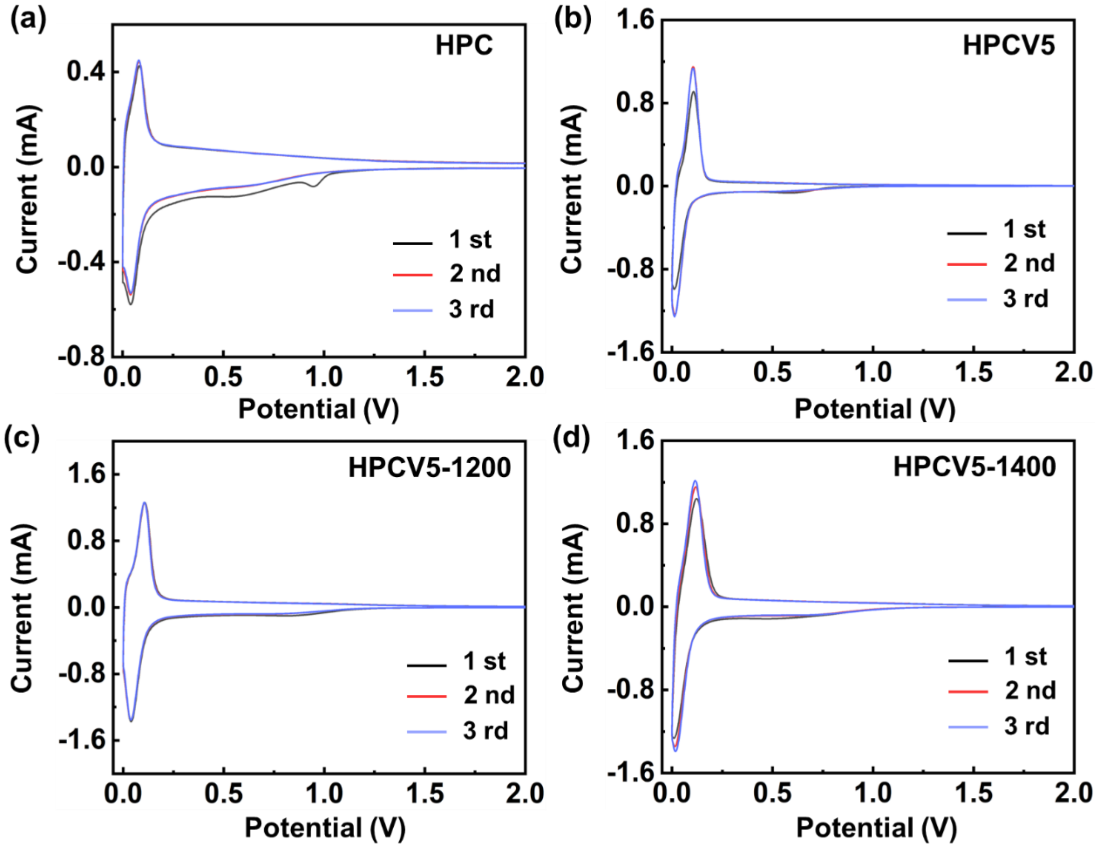

Figure S20. CV curves of a) HPC, b) HPCV5, c) HPCV5-1200 and d) HPCV5-1400 at 0.2 mV s^{-1}.

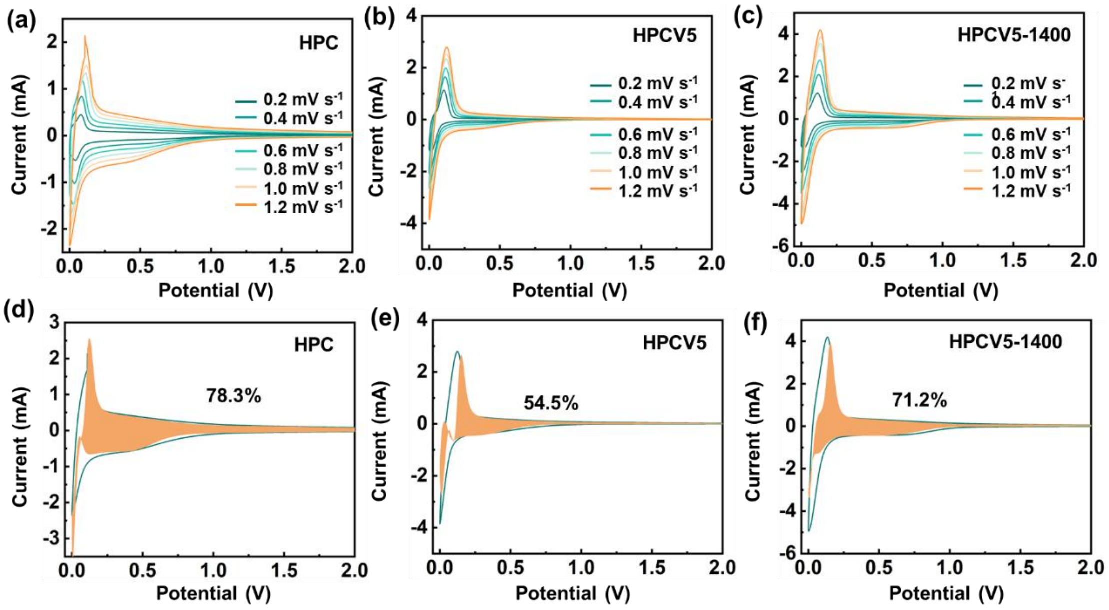

Figure S21. CV curves of a) HPC, b) HPCV5 and c) HPCV5-1400 at different scan rates. Capacitive contribution of d) HPC, e) HPCV5 and f) HPCV5-1400 at 1.2 mV s^{-1}.

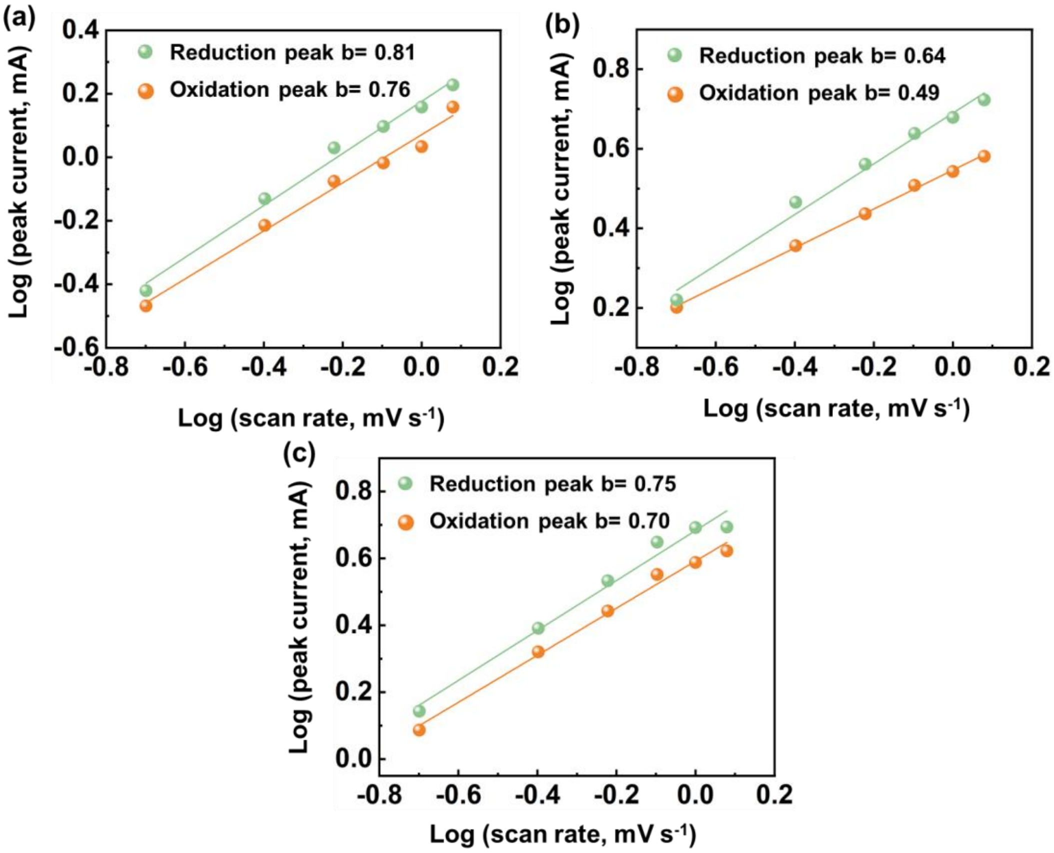

Figure S22. The corresponding correlations between peak current (i) and scan rate (mV s^{-1}) of a) HPC, b) HPCV5 and c) HPCV5-1400.

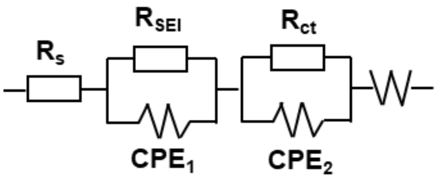

Figure S23. Equivalent electrical circuit model. (R_s represents the resistances associated with solution, wires, and contacts; R_{SEI} and CPE_1 (constant phase element) represent the Faradaic and non-Faradaic pathways of the SEI layer; R_{ct} is the charge transfer resistance, together with another CPE_2, represent the charge transfer reaction. The Warburg element W stands for the diffusion kinetics of the Na ion through the electrode materials.)

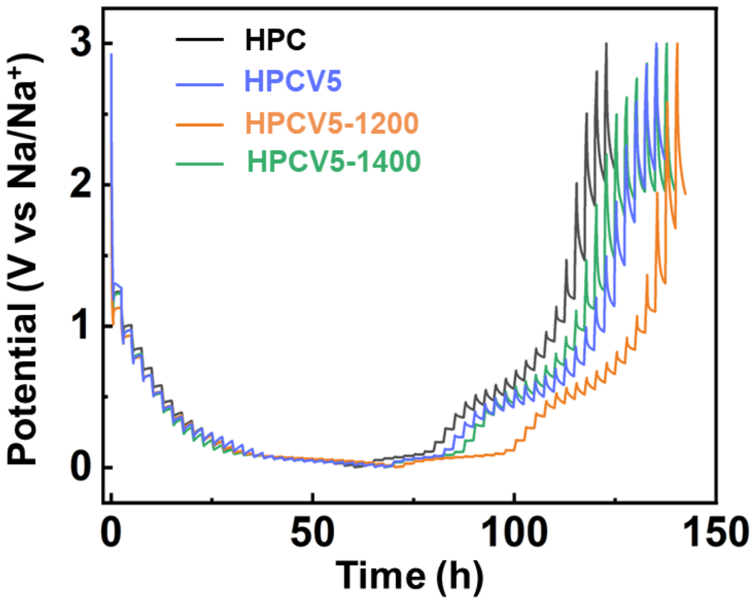

Figure S24. GITT curves of HPC, HPCV5, HPCV5-1200 and HPCV5-1400.

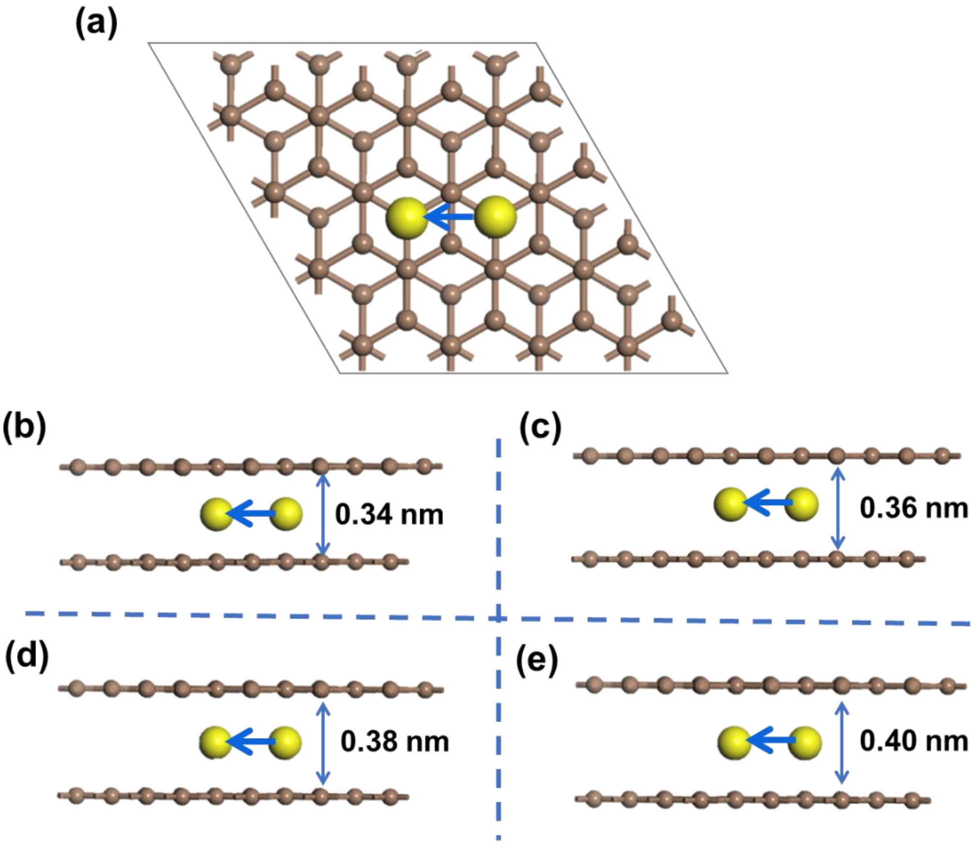

Figure S25. a) The top view of Na^+ migration pathways in graphitic layers. The side view of Na^+ migration pathways in graphitic layers with different interlayer distances of b) 0.34 nm, c) 0.36 nm, d) 0.38 nm and e) 0.40 nm.

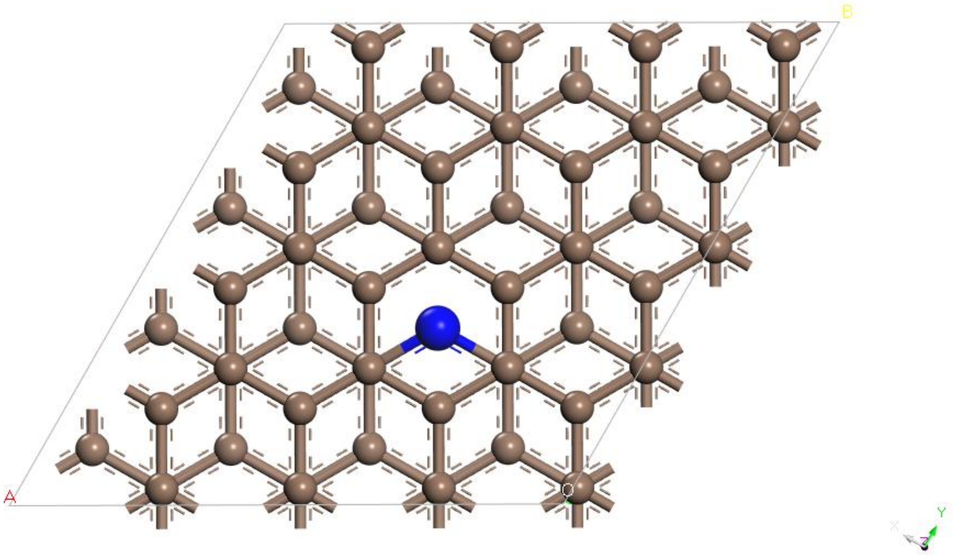

Figure S26. Optimization structure of the C-O-C oxygen defect in a bilayer graphene model with 0.38 nm interlayer spacing.

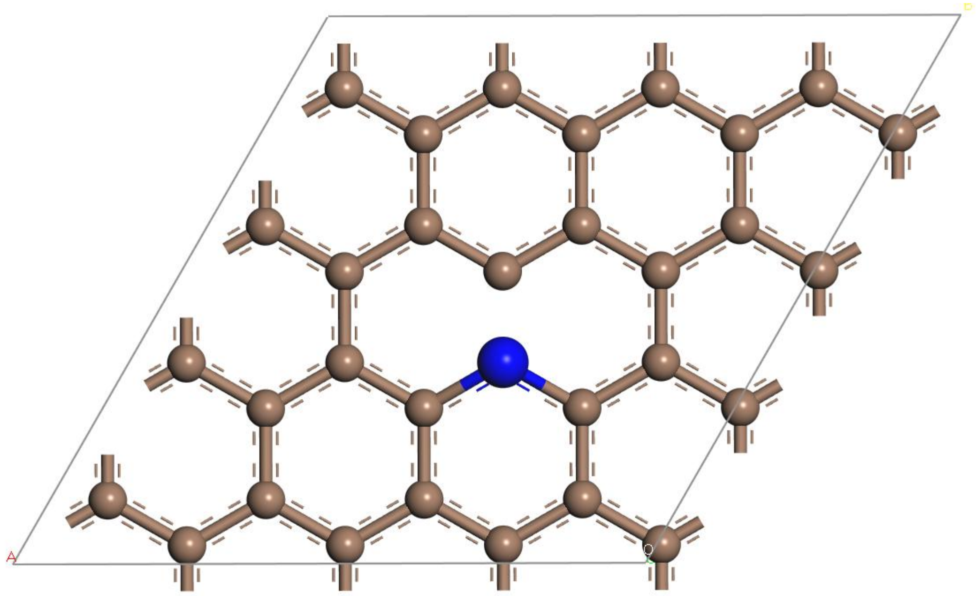

Figure S27. Optimized structures of graphene with C-O-C oxygen defects for the electrostatic potential map.

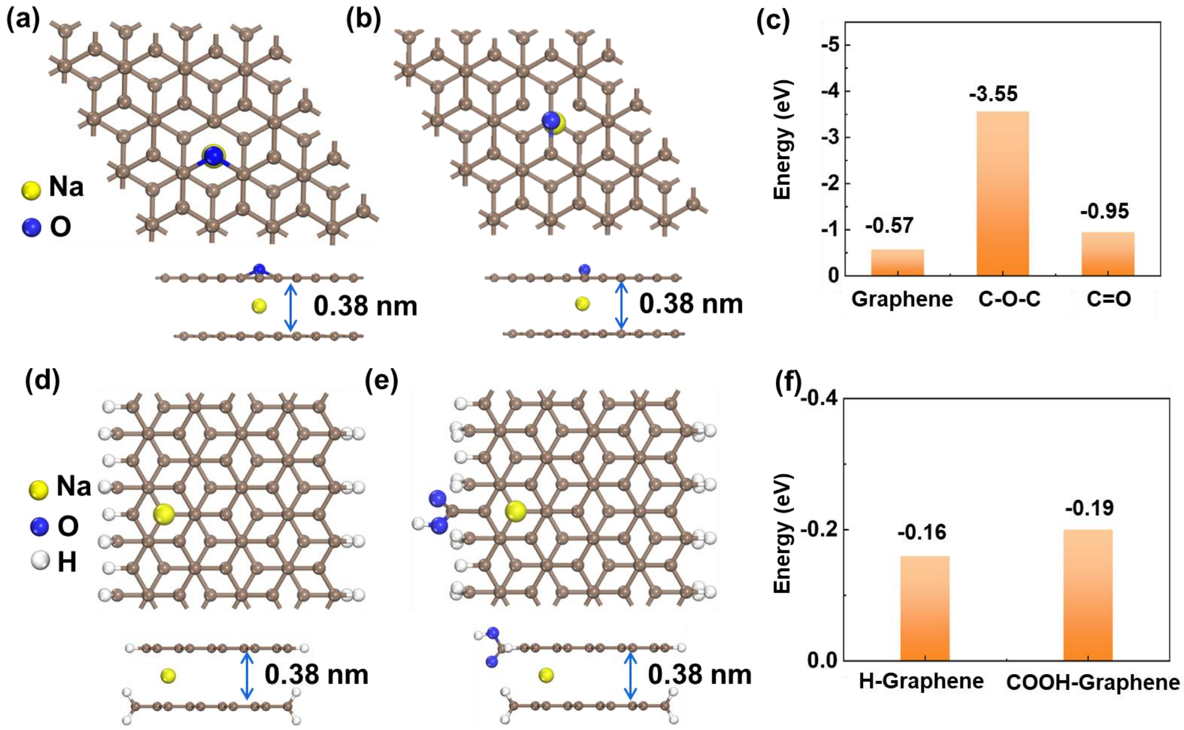

Figure S28. The model structure of a) C-O-C defective graphene, b) C=O defective graphene. c) The adsorption energy (E_a) of different model. The model structure of d) H-graphene, e) COOH-graphene. f) The Na^+ adsorption energy (E_a) of different model.

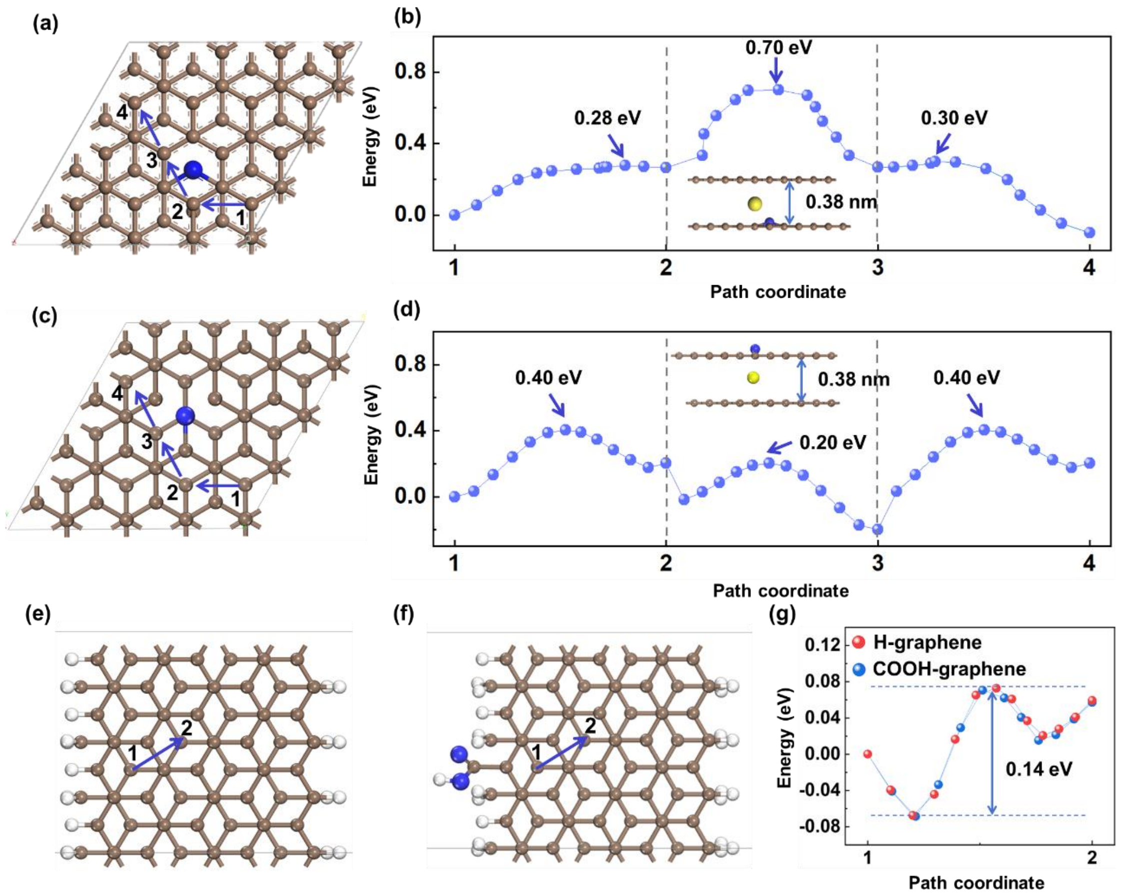

Figure S29. The model structure of the Na^+ migration pathways in a) C-O-C defective graphene, c) C=O defective graphene. The max Na^+ migration barriers of b) C-O-C defective graphene and d) C=O defective graphene. The model structure of the Na^+ migration pathways in e) H-graphene, f) COOH-graphene. g) The max Na^+ migration barriers of H-graphene and COOH-graphene.

Figure S28a, b show the models of bilayer graphene with C-O-C defects and with C=O defects, respectively. As shown in Figure S28c, the introduction of C-O-C and C=O into bilayer graphene leads to a significant increase in the Na^+ adsorption energy. The Na^+ adsorption energy for C-O-C defective graphene reaches -3.55 eV, which also means that a large force is required to drag Na^+ out from the "traps", hindering the rapid intercalation and extraction of Na^+.[7] Since -COOH groups typically appear at the edges of carbon materials, our work further complements the models of hydrogen-rich on the edges of the bilayer graphene (H-graphene) and the bilayer

graphene containing -COOH on its edges (COOH-graphene). As shown in Figure S28e-g, although the adsorption energy of Na^+ on COOH-graphene is essentially the same as that on H-graphene, it should also be noted that the -COOH groups can irreversibly react with Na^+ , thereby leading to a decrease in ICE.[8] Due to the excessively large Na^+ adsorption energy for C-O-C defective graphene, the max migration barriers of Na^+ reaches 0.70 eV, which is significantly higher than that for graphene (see Figure 5k, l of the manuscript). However, C=O defective graphene possesses a more suitable adsorption energy, resulting in a migration barriers that increases only from 0.26 eV to 0.40 eV. As shown in Figure S29, the Na^+ adsorption energy and migration barriers for the COOH-graphene and H-graphene are completely consistent, indicating that edge-COOH has almost no additional obstructive effect on the bulk migration of Na^+.

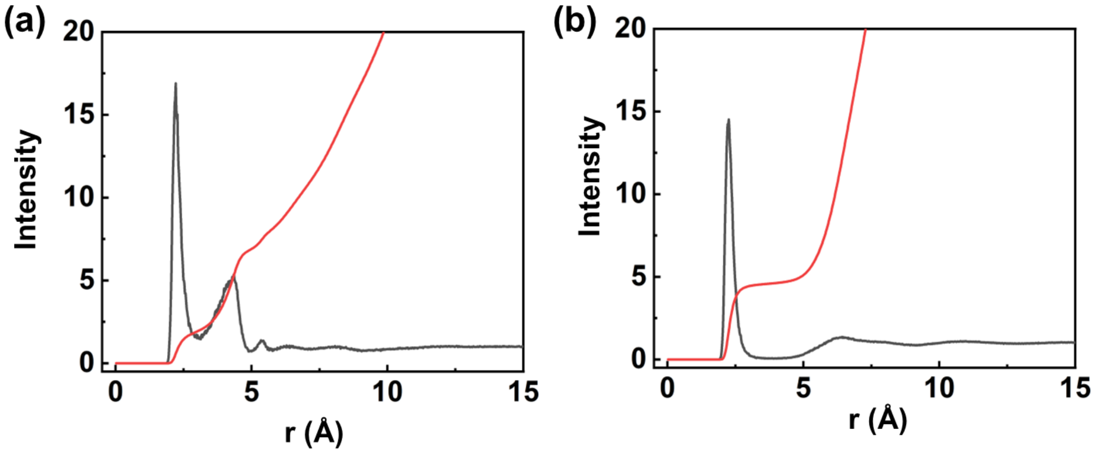

Figure S30. The radial distribution function of Na^+ to a) F or b) O ligands for 1 M $NaPF_6$.

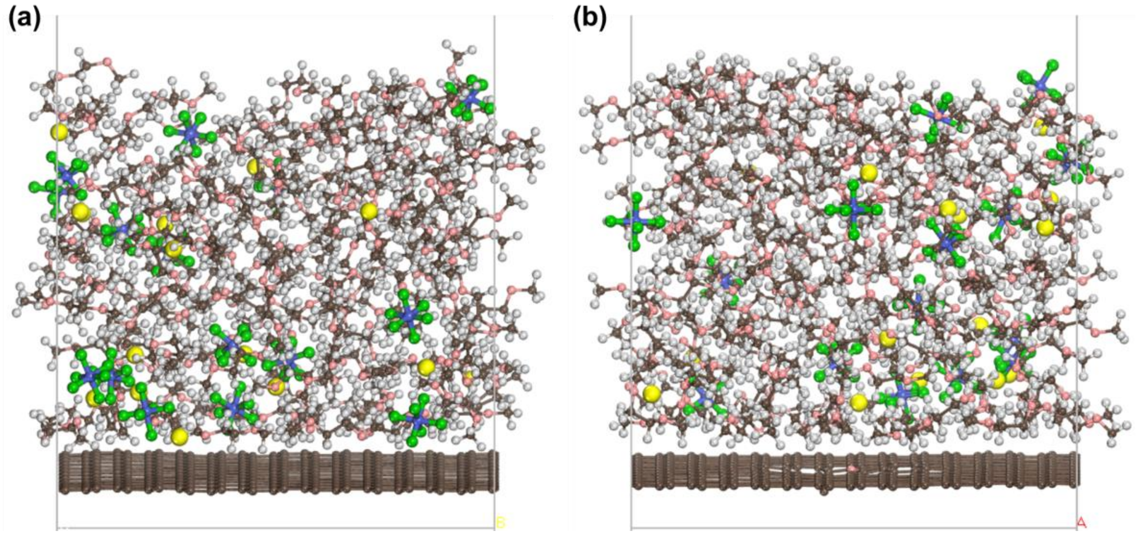

Figure S31. Models of electrolyte/HC interface for a) graphene and b) O-graphene.

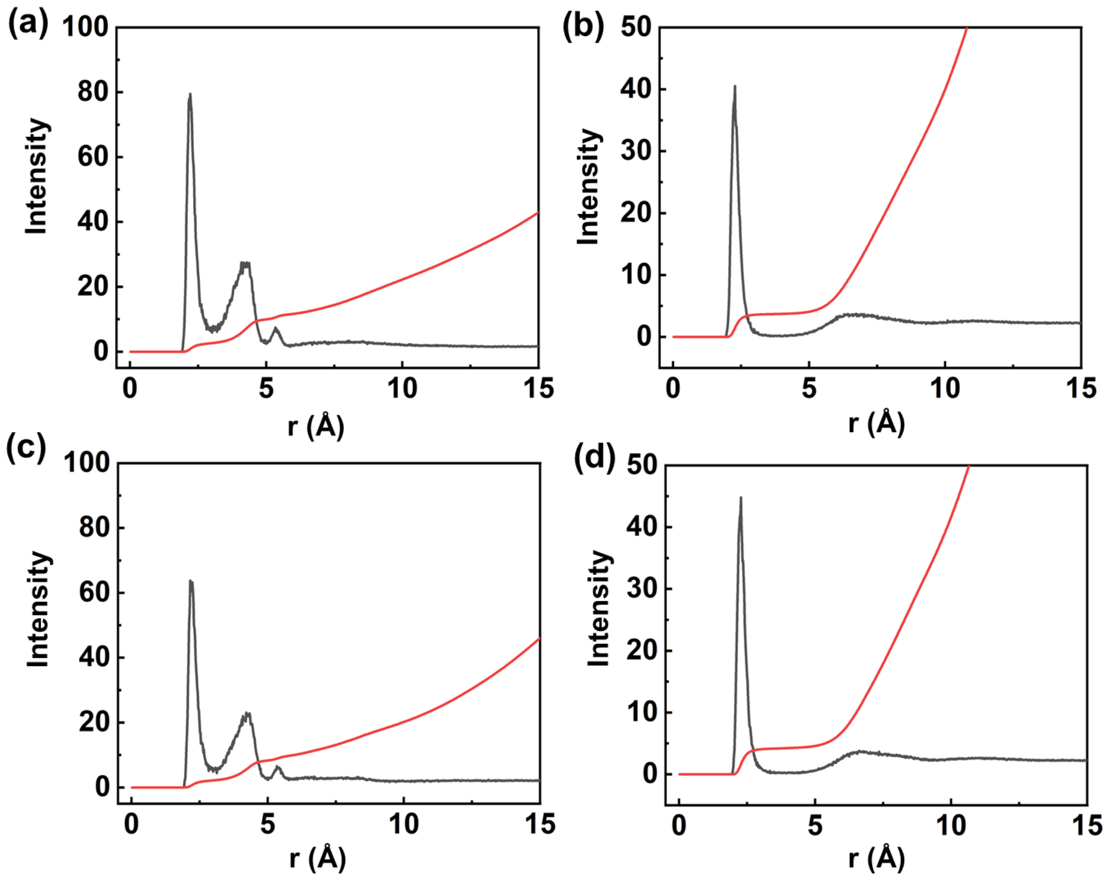

Figure S32. The radial distribution function of Na^+ to a) F or b) O ligands for graphene and Na^+ to c) F or d) O ligands for O-graphene models.

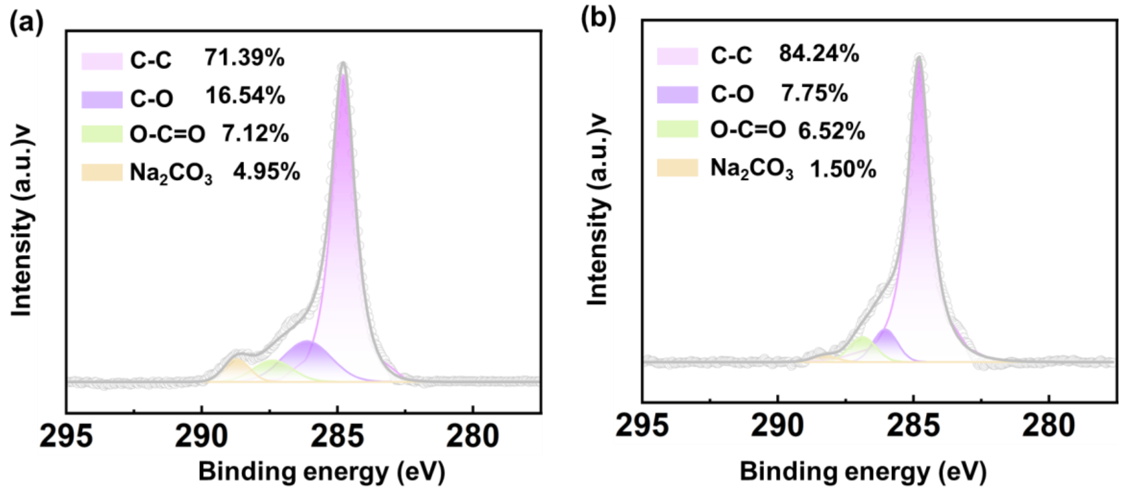

Figure S33. The high-resolution C 1 s spectra of a) HPCV5 and b) HPCV5-1200.

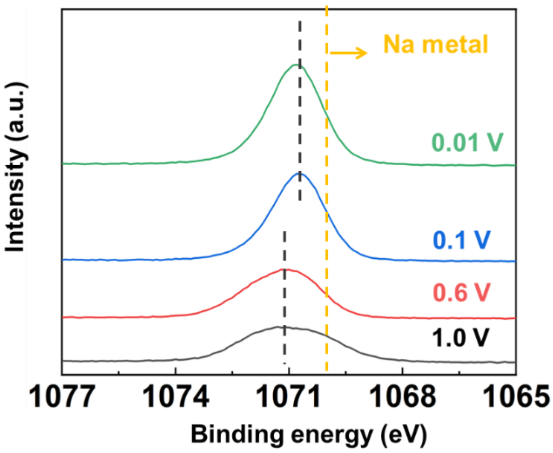

Figure S34. The high-resolution Na 1s spectra of HPCV5-1200 at different voltages.

As shown in Figure S34, the changes observed in high-resolution spectra of Na 1s can provide further valuable insights into the energy storage process. At the beginning, when the HPCV5-1200 electrode is discharged from 1.0 to 0.6 V, there is a notable increase in peak intensity without any shift in position, signifying the Na^+ adsorption within this voltage range. While further discharge to 0.1 V, it reveals an increase in peak intensity along with a gradual shift toward the characteristic peak of metallic sodium, this confirms the Na^+ intercalation behavior within the 0.6–0.1 V voltage range.[9] Notably, no further peak shift occurred in the plateau voltage region, indicating sodium filling into closed pores.

Table S1. Pore structure parameters of coal-tar-pitches based hard carbon samples.

Samples	S_{BET} (m^2 g^{-1})	$V_{total\ pore}$ (cm^3 g^{-1})	True density (g cm^{-3})	Closed pore volume (cm^3 g^{-1})
**HPC**	271.3	0.301	1.80	0.113
**HPCV5**	30.6	0.037	1.73	0.14
**HPCV5-1200**	9.6	0.034	1.68	0.153
**HPCV5-1400**	8.0	0.034	1.65	0.164

Table S2. Elemental analysis of all samples

Samples	C (wt%)	N (wt%)	S (wt%)	O (wt%)
HPC	96.25	0.21	0.02	2.13
HPCV5	95.90	0.15	0.03	2.61
HPCV5-1200	97.01	0.13	0.04	1.72
HPCV5-1400	97.91	0.10	0.02	0.96

Table S3. Comparison between HPCV-1200 in this work and previous reported hard carbon electrodes for sodium storage.

References	Precursor	Reversible capacity (mAh g^{-1}@A g^{-1})	Cycle number	Capacity after cycles (mAh g^{-1}@A g^{-1})	ICE	Publish year
This work	Pitch	284.5@0.2<br>262.3@0.5<br>229.3@2.0	300<br>800	320.6@0.2<br>192.6@2.0	91.6%	
10	Gluconate	~280@0.2<br>~260@0.5<br>~210@2.0	100	294.2@0.2	81.6%	2024
11	Glucose/ptich	258.6@0.2<br>192.5@0.5<br>69.1@2.0	120<br>500	313.3@0.02<br>250.6@0.2	92.08%	2025
[15]	Polystyrene	240.3@0.2<br>185@0.5<br>83.1@2.0	500	157.9@1.0	70.2%	2023
[16]	Tannin extract	~260@0.2<br>~230@0.5<br>~80@2.0	500	169.2@1.0	82.08%	2024
[17]	Olive shells	~230@0.2<br>~140@0.5<br>~70@2.0	100<br>1000	310@0.05<br>87@1.0	78%	2024
[18]	Carbon dots	~175@0.2<br>~100@0.5<br>~60@2.0	150	~220@0.1	68.4%	2023
[19]	Sisal hemp	~230@0.2<br>~180@0.5	50<br>1000	308@0.03<br>237.3@0.3	~63%	2024
[17]	Pitch	~100@0.2<br>~50@0.5	100	106@0.02	~82.82%	2023
18	Resin/pitch	~250@0.2<br>~130@0.5	100	359.8@0.03	74.8%	2024
19	Coal/pitch	~265@0.2<br>188@0.5	1000	230.5@0.3	88.4%	2023
20	Coal/sucrose	207@0.2<br>139@0.5	100<br>500	307@0.05	82.9%	2022
21	Platanus flosses	268@0.2<br>235@0.5<br>138@2.0	100<br>600	280@0.1<br>199@0.5	80.4%	2024

Table S4. The fitting data of impedance.

**Samples**	**R_{SEI}**	**R_{ct}**
**HPC**	4.32	27.5
**HPCV5**	1.84	7.04
**HPCV5-1200**	0.46	1.81
**HPCV5-1400**	1.30	5.18

References

[1] F. Xu, C.-Z. Qu, Q.-Q. Lu, J.-S. Meng, X.-H. Zhang, X.-S. Xu, Y.-Q. Qiu, B.-C. Ding, J.-Y. Yang, F.-R. Cao, P.-H. Yang, G.-S. Jiang, S. Kaskel, J.-Y. Ma, L. Li, X.-C. Zhang, H.-Q. Wang, *Sci. Adv.* **2022**, *8*, 7489.

[2] D. Sun, B. Luo, H.-Y. Wang, Y.-G. Tang, X.-B. Ji, L.-Z. Wang, *Nano Energy* **2019**, *64*, 103937.

[3] H.-Y. Fang, S.-N. Gao, M. Ren, Y.-H. Huang, F.-Y. Cheng, J. Chen, F.-J. Li, *Angew. Chem. Int. Ed.* **2023**, *62*, 202214717.

[4] X. Yin, Z. Lu, J. Wang, X. Feng, S. Roy, X. Liu, Y. Yang, Y. Zhao, J. Zhang, *Adv. Mater.* **2022**, *34*, 2109282.

[5] S.-W. Zhao, F.-Q. Huang, *ACS Nano* **2024**, *18*, 1733.

[6] H. Gao, L. Ding, H. Bai, A.-H. Liu, S.-Z. Li, L. Li, *J. Mater. Chem. A* **2016**, *4*, 16490.

[7] N. Sun, B. Luo, H.-Y. Wang, Y.-G. Tang, X.-B. Ji. L.-Z. Wang, Nano Energy **2019**, *64*, 103937.

[8] R.-B. Dang, Y.-X. Lu, X.-H. Rong, F.-X. Ding, Q.-B. Guo, W.-L. Xu, L.-Q. Chen, Y.-S. Hu, *Chin. Sci. Bull.* **2022**, *67*, 3546.

[9] Y.-J. Huang, X.-Y. Hu, Y.-J. Li, X. Zhong, Z.-D. He, Z.-L. Geng, S.-Y. Gan, W.-T. Deng, G.-Q. Zou, H.-S. Hou, X.-B. Ji, *Adv. Funct. Mater.* **2024**, *34*, 2403648.

[10] H.-L. Sun, Q.-Y. Zhang, Y.-Q. Ma, Z.-J. Li, D. Zhang, Q.-J. Sun, Q.-J. Wang, D. Liu, B. Wang, *Energy storage Mater*. **2024**, *67*, 103269.

[11] Y.-L. Ji, S.-Q. Li, T. Yuan, Q.-H. Shi, X.-H. Hu, Q.-S. Shao, W.-L. Feng, Y.-F, Zhao, *J. Colloid Interface Sci.* **2025**, *677*, 719.

[12] Y.-Q. Qiu, G.-S. Jiang, Y.-X. Su, X.-R. Zhang, Y.-X. Du, X.-S. Xu, Q. Ye, J.-B. Zhang, M.-H. Ban, F. Xu, H.-Q. Wang, *Carbon Energy.* **2024**, *6*, 479.

[13] Q. Hu, L.-Q. Xu, G.-G. Liu, J.-B. Hu, X.-B. Ji, Y.-Q. Wu, *ACS Nano* **2024**, *18*, 21491.

[14] L. Zhou, Y.-P. Cui, P.-C. Niu, L.-N. Ge, R.-M. Zheng, S.-H. Liang, W. Xing, *Carbon* **2024**, *231*, 119733.

[15] Y.-J. Huang, X. Zhong, X.-Y. Hu, Y.-J. Li, K. Wang, H.-Y. Tu, W.-T. Deng, G.-Q. Zou, H.-S. Hou, X.-B. Ji, *Adv. Funct. Mater*. **2023**, *34*, 2308392.

[16] H.-H. Ou, B.-Y. Pei, Y.-F. Zhou, M. Yang, J.-N. Pan, S.-Q. Liang, X.-X. Cao. *Small Methods*.

2024, *9*, 2400839.

[17] R. Xu, Z.-L. Yi, M.-X. Song, J.-P. Chen, X.-X. Wei, F.-Y. Su, L.-Q. Dai, G.-H. Sun, F. Yang, L.-J. Xie, C.-M. Chen, *Carbon* **2023**, *206*, 94.

[18] D. Sun, L. Zhao, P.-L. Sun, K. Zhao, Y.-K. Sun, Q. Zhang, Z.-C. Li, Z. Ma, F.-Z. Zheng, Y. Yang, C.-B. Lu, C. Peng, C.-M. Xu, Z.-H. Xiao, X.-L. Ma, *Adv. Funct. Mater.* **2024**, *34*, 2403642.

[19] H. Chen, N. Sun, Y.-X. Wang, R. A. Soomro, B, Xu, *Energy Storage Mater.* **2023**, *56*, 532.

[20] H. Chen, N. Sun, Q.-Z. Zhu, R.-Z. Ali Soomro, B. Xu, *Adv. Sci*. **2022**, *9*, 2200023.

[21] Z.-D. Hou, M.-W. Jiang, D. Lei, X. Zhang, Y.-Y. Gao, J.-G. Wang, *Nano Research* **2024**, *17*, 5188.
